# Dissociations in semantic cognition: Oscillatory evidence for opposing effects of semantic control and type of semantic relation in anterior and posterior temporal cortex

**DOI:** 10.1016/j.cortex.2019.07.002

**Published:** 2019-11

**Authors:** Catarina Teige, Piers L. Cornelissen, Giovanna Mollo, Tirso Rene del Jesus Gonzalez Alam, Kristofor McCarty, Jonathan Smallwood, Elizabeth Jefferies

**Affiliations:** a Department of Psychology and York Neuroimaging Centre, University of York, UK; b Department of Psychology, Northumbria University, UK

**Keywords:** MEG, Semantic, Anterior temporal lobe, Posterior middle temporal gyrus, Angular gyrus, Controlled retrieval

## Abstract

How does the brain represent and process different types of knowledge? The Dual Hub account postulates that anterior temporal lobes (ATL) support taxonomic relationships based on shared physical features (mole – cat), while temporoparietal regions, including posterior middle temporal gyrus (pMTG), support thematic associations (mole – earth). Conversely, the Controlled Semantic Cognition account proposes that ATL supports both aspects of knowledge, while left pMTG contributes to controlled retrieval. This study used magnetoencephalography to test these contrasting predictions of functional dissociations within the temporal lobe. ATL and pMTG responded more strongly to taxonomic and thematic trials respectively, matched for behavioural performance, in line with predictions of the Dual Hub account. In addition, ATL showed a greater response to strong than weak thematic associations, while pMTG showed the opposite pattern, supporting a key prediction of the Controlled Semantic Cognition account. ATL showed a stronger response for word pairs that were more semantically coherent, either because they shared physical features (in taxonomic trials) or a strong thematic association. These effects largely coincided in time and frequency (although an early oscillatory response in ATL was specific to taxonomic trials). In contrast, pMTG showed non-overlapping effects of semantic control demands and thematic judgements: this site showed a larger oscillatory response to weak associations, when ongoing retrieval needed to be shaped to suit the task demands, and also a larger response to thematic judgements contrasted with taxonomic trials (which was reduced but not eliminated when the thematic trials were easier). Consequently, time-sensitive neuroimaging supports a complex pattern of functional dissociations within the left temporal lobe, which reflects both coherence versus control and distinctive oscillatory responses for taxonomic overlap (in ATL) and thematic relations (in pMTG).

## 1 Introduction

Although the network of brain regions supporting semantic cognition is well-established (Binder et al., 2009, Lambon Ralph et al., 2017), it is unclear whether functional distinctions between these sites reflect differences in *content* or *process* (Mirman, Landrigan, & Britt, 2017). Content-based accounts suggest that different brain regions represent distinct types of knowledge (de Zubicaray et al., 2013, Schwartz et al., 2011). For example, taxonomic relationships (e.g., the link between dog and mouse, which share physical features) might be represented in the anterior temporal lobes (ATL), while thematic relationships (e.g., knowledge that dog and leash are found together) might be maintained by temporoparietal regions. Other frameworks propose a single semantic store, which encompasses these different aspects of knowledge (e.g., the Controlled Semantic Cognition model; Lambon Ralph et al., 2017). According to this view, functional differences between heteromodal semantic sites reflect the extent to which tasks require the engagement of control processes to shape retrieval. This study seeks to reconcile these contrasting frameworks by using a temporally sensitive neuroimaging method (magnetoencephalography; MEG) to characterise how the evolving response to individual words is modulated by thematic and taxonomic relationships to a preceding word.

ATL is thought to integrate different sources of modality-specific information representing colour, shape, movement etc., to allow the computation of coherent heteromodal concepts (Lambon Ralph et al., 2017, Patterson et al., 2007, Rogers et al., 2006). Information integration in ATL is thought to be graded, with the most heteromodal response in middle and inferior temporal gyri, and a stronger response to verbal and auditory inputs in anterior superior temporal gyrus (aSTG; Murphy et al., 2017, Visser et al., 2012). Information about where objects are found and how they are used could be integrated along with physical properties in a single semantic hub in ATL (Hoffman et al., 2018, Jackson et al., 2015). Alternatively, the Dual Hub framework proposes that ATL underpins taxonomic knowledge while temporoparietal areas, such as posterior middle temporal gyrus (pMTG) and angular gyrus (AG), extract event associations and thematic knowledge (de Zubicaray et al., 2013, Schwartz et al., 2011). This perspective was originally motivated by neuropsychological research showing that patients with lesions in temporoparietal areas make more thematic errors in picture naming (e.g., dog → bone), while those with lesions in ATL produce more categorical errors (e.g., dog → cat) (Schwartz et al., 2011). However, thematic errors (such as responding ‘leash’ to a picture of a dog) might also imply the *preservation* of semantic information but difficulty tailoring retrieval to suit the demands of the task (Jefferies & Lambon Ralph, 2006).

Neuroimaging evidence has also implicated temporoparietal areas in the retrieval of knowledge about thematic relations and events, in line with the Dual Hub account, although there is some diversity across studies, with activation peaks in pMTG, AG and superior temporal sulcus (Bedny et al., 2014, Kalenine et al., 2009, Sass et al., 2009, Schwartz et al., 2011). Conversely, taxonomic relations elicit stronger recruitment of visual regions, potentially reflecting the visual similarity of objects within categories (Kalenine et al., 2009, Kotz et al., 2002). ATL falls at the end of the ventral visual stream (Clarke et al., 2013, Clarke et al., 2011), consistent with the claim that ATL supports taxonomic knowledge. However, studies observing this dissociation in conceptual representation have often failed to match the difficulty of taxonomic and thematic judgements, potentially contributing to differences in peak activations between studies (Sachs et al., 2008a, Sachs et al., 2008b, Sass et al., 2009). Moreover, a recent fMRI study identified a common response to categorical and thematic relationships throughout the semantic network when difficulty was controlled in the analysis (Jackson et al., 2015).

The importance of task difficulty is emphasised in an alternative theoretical account – the Controlled Semantic Cognition framework – which suggests that while ATL acts as a long-term semantic store, left pMTG, together with inferior frontal gyrus (IFG), supports the controlled retrieval of conceptual information (Jefferies, 2013, Lambon Ralph et al., 2017). Both left pMTG and IFG show a stronger response when non-dominant information must be brought to the fore, for example when participants retrieve weak associations, non-dominant interpretations of ambiguous words and targets presented with strong distractors (Davey, Cornelissen et al., 2015, Noonan et al., 2013, Badre et al., 2005). Inhibitory TMS to pMTG and IFG produces equivalent disruption of weak but not strong semantic associations, suggesting both of these sites play a critical role in semantic control (Whitney, Kirk, O'Sullivan, Lambon Ralph, & Jefferies, 2011). Moreover, they show strong intrinsic connectivity at rest, and disruption of left IFG elicits compensatory increases in activity in pMTG (Hallam, Whitney, Hymers, Gouws, & Jefferies, 2016), consistent with their participation in a large-scale network for semantic control. Importantly, both of these sites lie outside the ‘multiple-demand’ system that responds to executive control demands across domains (Duncan, 2010). This might be because in many tasks tapping semantic control, there is no explicit or externally-presented goal which specifies the aspects of concepts that should be prioritised at a given moment – instead, the combination of concepts themselves determines the semantic control demands. When one word sets up a pattern of semantic retrieval which is highly relevant to understanding the conceptual link with a second word (i.e., the meaning of the two words is highly coherent), control demands are thought to be minimised, because the pattern of conceptual retrieval within the semantic store does not need to be substantially altered. In contrast, when participants are required to understand a conceptual link between two words that are only weakly related (e.g., cushion-cat in the weak thematic condition), it is necessary to guide retrieval away from highly related but irrelevant concepts such as fabric and dog and focus on specific information that links cats to cushions (Davey et al., 2016).

In summary, the Dual Hub account suggests that ATL represents taxonomic knowledge while pMTG (and AG) represent thematic knowledge (e.g., Schwartz et al., 2011). In contrast, the Controlled Semantic Cognition Framework proposes that ATL represents all aspects of semantic knowledge, while pMTG (and IFG) support controlled semantic retrieval (Hoffman et al., 2018, Jefferies, 2013, Lambon Ralph et al., 2017). To reconcile these opposing accounts of the functional organisation of conceptual processing, the current study contrasted (i) taxonomic and thematic judgements matched for difficulty according to behavioural performance, and (ii) judgements about strong and weak thematic associations selected to have varying control demands. We used magnetoencephalography (MEG) combined with a paradigm in which pairs of words were presented individually, to characterise neural recruitment through time. fMRI may lack sensitivity to differences between different patterns of semantic retrieval, since its slow time-course prevents the separation of semantic retrieval to each meaningful item (which would be similar across the semantic network irrespective of the merits of the different theoretical accounts) from the modulation of this response according to the semantic relationship between items. There have been very few MEG studies of taxonomic and thematic decisions (Lewis, Poeppel, & Murphy, 2015), and none that have manipulated both semantic content and control requirements. Consequently, the current study provides a unique characterisation of functional dissociations within the semantic system.

We tested whether the functional distinction between ATL and pMTG is best characterised in terms of type of relationship (taxonomic *vs* thematic, as anticipated by the Dual Hub theory) or controlled retrieval demands (e.g., the contrast of more related *vs* less related words, as anticipated by the Controlled Semantic Cognition framework). To anticipate, we observed both of these patterns. We found that ATL is sensitive to both the overlap of physical features that tend to be shared across taxonomically-related items (taxonomic > thematic judgements), and to the strength of co-occurrence for concepts that are found or used together (strong > weak thematic). pMTG showed the opposite pattern: this site responded more strongly to thematic trials relative to taxonomic trials, irrespective of difficulty, and also to weak compared with strong thematic trials, when demands on controlled retrieval are thought to be increased.

## 2 Methods

### 2.1 Participants

We report how we determined our sample size, all data exclusions, all inclusion/exclusion criteria, whether inclusion/exclusion criteria were established prior to data analysis, all manipulations, and all measures in the study. Participants were 20 right-handed native English speakers, with normal or corrected-to-normal vision, and no history of language disorders (6 males, mean age 26.7, range 18–37, with these inclusion criteria established prior to data collection). This sample size was selected in line with other similar studies. The study was conducted in accordance with the Research Ethics and Governance Committee of the York Neuroimaging Centre, University of York, UK, and written informed consent was obtained. One participant was excluded from the analysis because their accuracy on catch trials was poor (less than 75%).

### 2.2 Materials

There were three experimental conditions: strong thematic associations, weak thematic associations, and taxonomically related word pairs. These conditions permitted a comparison of thematic and taxonomic trials – both when these trials were matched for difficulty (measured in terms of rated difficulty, and behavioural performance, in the contrast weak thematic *vs* taxonomic) and when the taxonomic trials were harder (strong thematic *vs* taxonomic). We were also able to compare thematic associations differing in difficulty (weak *vs* strong associations). These contrasts tested the key predictions of both the Dual Hub and the Controlled Semantic Cognition accounts. To select the stimuli, participants who did not take part in the MEG experiment (n = 30) were asked to rate word pairs on three questions probing 1: Thematic relatedness (co-occurrence): “How associated are these items? For example, are they found or used together regularly?”; 2: Taxonomic relatedness (physical similarity): “Do these items share similar physical features?” and 3: “How easy overall is it to identify a connection between the words?”. Ratings were made on a Likert scale from 1 to 7 (1 = Not at all, 7 = Very). Selected word pairs were rated as highly similar on one type of relationship and not the other (see Table 1).

**Table 1:** TOP: means and standard deviations for rated co-occurrence (Q1), physical similarity (Q2) and difficulty (Q3). BOTTOM: *t*-tests between conditions.

CONDITION	Q1	Q2	Q3	Word2vec
Taxonomic				
Mean	3.10	5.00	4.51	.324
*SD*	.93	.81	.84	.122
Thematic strong				
Mean	6.46	1.49	6.33	.327
*SD*	.65	.74	.73	.124
Thematic weak				
Mean	5.42	1.66	4.52	.242
*SD*	1.16	1.20	1.64	.129
*t*-tests	Q1	Q2	Q3	Word2vec
Taxonomic *vs* Thematic strong	*p* < .001	*p* < .001	*p* < .001	n.s.
Taxonomic *vs* Thematic weak	*p* < .001	*p* < .001	n.s.	*p* < .001
Thematic strong *vs* Thematic weak	*p* < .001	n.s.	*p* < .001	*p* < .001

Q1: Rated co-occurrence. Q2 = Rated physical similarity. Q3 = Rated difficulty.

We also extracted word2vec scores (Mikolov, Chen, Corrado, & Dean, 2013), as a global measurement of the semantic similarity of the pairs of words in each condition (see Table 1). Word2vec captures similarities in the meanings of words based on similarities between the linguistic contexts in which they are used. In this way, word2vec scores are sensitive to both taxonomic and thematic relations. Items with physical similarities can be described in similar ways, even if they do not often co-occur in the same context (for example, cow and bear both feed, walk, car for their young etc.). Similarly, items that are thematically related also co-occur with a shared set of words (for example, bib and child both co-occur with milk and dummy etc., even though they share no physical features). The Controlled Semantic Cognition account proposes that ATL extracts conceptual knowledge from the sum of all our experiences – and the similar linguistic contexts seen for items with overlapping physical features and strong thematic links can provide a proxy for this. A comparison of word2vec across conditions found higher semantic similarity for strong versus weak thematic trials, and also for taxonomic versus weak thematic trials, even though these conditions were matched on behavioural performance and rated difficulty (see Table 1). Moreover, the taxonomic versus strong thematic trials were matched on word2vec, yet the strong thematic trials were easier as measured by behavioural performance and rated difficulty.

The stimuli are provided in the online supplementary information. The ratings and word2vec scores are provided on Open Science Framework (osf.io/mz52c).

### 2.3 Procedure

We presented 95 target words in the three semantically-related conditions: the first word in the pair differed across conditions, while the second word – the target triggering the semantic decision – was the same across conditions (see supplementary materials, which provides a full list of items). This ensured that the visual and lexical features of the stimuli being compared were the same. Any differences in response to the target across conditions therefore reflected the pattern of retrieval needed to form a meaningful link between the second word and the first word. There were 95 taxonomically-related primes, 95 strongly thematically associated primes and 95 weakly thematically associated primes, alongside 100 unrelated trials, which presented the same 95 target words, plus 5 additional targets.

**Fig. 1:**
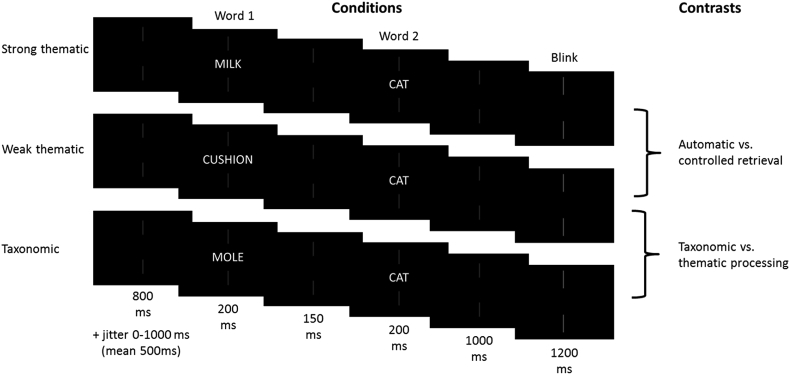
Illustration of strong thematic, weak thematic and taxonomic trials. The words are not to scale; for visibility they have been made larger and brighter.

Each pair was presented one word at a time. Nonius lines (acting as a fixation cross; see Fig. 1) were present at all times. Before each trial, there was a rest period of 800 ms, plus an unpredictable jittered interval from 0 to 1000 ms (mean 500 ms), designed to reduce anticipatory responses. The first word in the pair was presented for 200 ms, there was an inter-stimulus interval (ISI) of 150 ms, and then the second word appeared for 200 ms followed by a 1000 ms interval. A short ISI has been shown to produce priming effects for both thematic and taxonomic association (Jones & Golonka, 2012). After each trial, the nonius lines changed to a dimmer red (for 1000 ms) and participants were encouraged to confine blinking/swallowing to this period. An illustration of the trial structure can be seen in Fig. 1. On an additional 10% of trials, participants were cued to make an overt response by the presence of a question mark (on screen for 1000 ms) after the target presentation. They pressed one of two buttons with their left hand to indicate if the two words were related. These ‘catch trials’ were used to monitor performance in the task, and were disregarded from the MEG analysis.

The stimuli were presented within three equal-length blocks, containing all trial types. The order of these blocks was counterbalanced between participants.

### 2.4 Stimulus presentation

Presentation version 16.1 (Neurobehavioral Systems) was used to present the stimuli and to record responses on catch trials. Stimuli were back-projected onto a screen with a viewing distance of ∼75 cm, so that letter strings subtended ∼1° vertically and ∼5° horizontally at the retina. We presented light grey letters on a dark grey background such that the screen luminance was in the mesopic range, and a neutral density filter was used to minimize glare.

### 2.5 Data collection

Before MEG data acquisition, participants' head shape and the location of five head coils were recorded with a 3D digitizer (Fastrak Polhemus). The head coils were used to localise the position of the head within the helmet before and after the experiment. For each participant, a high-resolution structural T1-weighted anatomical volume was acquired in a GE 3.0 T Signa Excite HDx system (General Electric, USA) at the York Neuroimaging Centre, University of York, with an 8-channel head coil and a sagittal-isotropic 3-D fast spoiled gradient-recalled sequence. The 3D digitized head shape of each participant was used for the co-registration of individual MEG data onto the participant's structural MRI image using a surface-based alignment procedure (Kozinska, Carducci, & Nowinski, 2001).

MEG data were collected in a magnetically shielded room, with participants seated in an upright position, using a whole-head 248-channel, Magnes 3600 (4D Neuroimaging, San Diego, California), with the magnetometers arranged in a helmet shaped array. Data were recorded in continuous mode, with a sampling rate of 678.17 Hz and pass-band filtered between 1 and 200 Hz. MEG signals were subjected to a global field noise filter subtracting external, non-biological noise detected by the MEG reference channels, and converted into epochs of 1500 ms length, starting 800 ms before the target onset. All channels from all trials were inspected visually in an artefact rejection process. Data from three noisy channels were automatically rejected. Additional trials were rejected if eye blinks, movement artefacts or external noise sources were evident. On average, 10.9% of trials were rejected (minimum 4.6%; maximum 25%).

The procedures were not pre-registered prior to the research being conducted.

### 2.6 MEG analysis

There were two stages to the analysis. We first characterised the response to semantically-related trials across the whole brain (collapsing the taxonomic, strong thematic and weak thematic conditions). This analysis examined the neural response in terms of total oscillatory power, at a coarse frequency and time resolution, to establish the location of peak responses within the semantic network. In a second phase of the analysis, we contrasted the response for taxonomic and weak thematic trials (matched for difficulty), taxonomic and strong thematic trials (where the taxonomic trials were more demanding), and strong versus weak thematic trials (differing in control demands but matched on semantic decision type). These contrasts were performed at a fine frequency and time resolution within Points of Interest (POI). The locations were selected on the basis of their importance to theories of semantic processing and defined with reference to peak responses in the whole-brain beamforming data.

For both whole-brain and POI analyses, the neural sources of the brain activity were reconstructed with a modified version of the vectorised, linearly-constrained minimum-variance (LCMV) beamformer (Huang et al., 2004, Van Veen et al., 1997) implemented in the Neuroimaging Analysis Framework pipeline (NAF, York Neuroimaging Centre), using a multiple spheres head model (Huang, Mosher, & Leahy, 1999), with co-registrations checked manually. An MEG beamformer (spatial filter) allows an estimation of the signal coming from a location of interest while attenuating the signal coming from other points in the brain. This is achieved by constructing the neuronal signal at a given point in the brain as the weighted sum of the signals recorded by the MEG sensors. The sensor weights were determined by an optimisation algorithm, whereby the signal was maximised from the location of interest, and minimised for other locations. Independent beamformers were reconstructed for each point in the brain, in each of three orthogonal current directions. The covariance matrix used to generate the weights of each beamformer was regularized using an estimate of noise covariance as described in Hymers, Prendergast, Johnson, and Green (2010) and Prendergast, Johnson, Hymers, Woods, and Green (2011). This procedure was performed separately for each condition and/or analysis window, in order to obtain an optimal sensitivity to the effect of interest (in line with Brookes et al., 2011, Brookes et al., 2008). The outputs of the three spatial filters at each point in the brain (referred to as a Virtual Electrode) were summed to generate estimates of oscillatory power. For the whole-brain analysis, a noise normalised volumetric map of total power (i.e., including both the evoked and non-phase locked components) was produced over a given temporal window and within pre-specified frequency bands. For the POI analysis, the time course information at the location specified was reconstructed and the time-frequency decomposition was computed using Stockwell Transforms (Stockwell, Mansinha, & Lowe, 1996), to obtain higher resolution in time and frequency. This analysis strategy and the parameters used for the current study were similar to those used in recent MEG studies of visual word recognition and object naming (Klein et al., 2014, Mollo et al., 2017, Mollo et al., 2018, Teige et al., 2018, Urooj et al., 2014, Wheat et al., 2010). The analysis pipeline is publically available (vcs.ynic.york.ac.uk/naf/naf). The conditions of our ethical approval do not permit public archiving of anonymised data because participants did not provide sufficient consent. Researchers who wish to access the data should contact the Research Ethics and Governance Committee of the York Neuroimaging Centre, University of York, or the corresponding author, Beth Jefferies. Sufficient data to replicate all results reported in the paper will be released to researchers, subject to the approval of the Research Ethics and Governance Committee of the York Neuroimaging Centre, University of York, when this is possible under the terms of the GDPR.

#### 2.6.1 Whole brain beamforming

The brain's response to semantically-related trials was characterised within broad frequency ranges and across 200 ms time periods (collapsing across taxonomic, strong thematic and weak thematic conditions). The purpose of this analysis was to identify local peaks in oscillatory power within theoretically-relevant brain regions, which were then investigated in more detail in a POI analysis (see below). Our research question concerned how the brain's response to the second word changed as a function of its relationship to the first word. We therefore analysed the whole-brain beamforming data by contrasting “active” and “passive” time windows of 200 ms duration from the onset of the second word (0–200 ms, 200–400 ms, and 400–600 ms). In the passive time window (−700 to −500 ms relative to the onset of the second word), participants observed the (always present) nonius (fixation) lines.

A 3D lattice of points was constructed across the whole brain with 5-mm spacing, and beamformers were used to compute the total power using the Neural Activity Index (Van Veen et al., 1997) – an estimate of oscillatory power that takes account of spatially-inhomogeneous noise – at each point independently, within the following frequency bands: 5–15 Hz, 15–25 Hz, 25–35 Hz and 35–50 Hz. These frequency ranges were taken from previous MEG studies of reading (Klein et al., 2014). In the whole-brain beamforming analyses, we examined total power, which combines evoked (phase-locked to the stimulus) and induced (non-phase locked) components, in each frequency band. For each individual participant and each frequency band, this analysis produced an NAI volumetric map for the two time-windows or conditions being compared. A paired-samples t-statistic was used to characterise the difference between these maps at each point in space. Individual participant's t-maps were transformed into standardized space and superimposed on the MNI template brain using MRIcroN software (Rorden, Karnath, & Bonilha, 2007).

In order to determine whether the difference between conditions or time-windows was statistically significant for each point on the lattice, we built up a null distribution by randomly relabelling the two time points for each participant and each voxel, using the permutation procedure developed by Holmes, Blair, Watson, and Ford (1996). We established the maximum t-value obtained with random relabelling across 10000 permutations. We then compared the real distribution of t-values in our data with the maximum t-value obtained from the permuted data. Maximum statistics can be used to overcome the issue of multiple comparisons (i.e., controlling experiment-wise type I error), since the approach uses the highest permuted t-value across the brain to provide a statistical threshold for the whole lattice of points, over which the null hypothesis can be rejected (Holmes et al., 1996). Fig. 3 shows those voxels in the brain with t-values equal or higher than the top 5% t-values present in the null distribution.

#### 2.6.2 Time-frequency analysis: point of interest (POI)

We placed POIs in brain regions (i) showing a strong oscillatory response in the whole-brain beamforming data across conditions and (ii) for which the Dual Hub and Controlled Semantic Cognition accounts make different predictions. Two temporal lobe sites met these requirements and are the focus of the analysis below. There was a local peak in left ATL, in the 200–400 ms time window and 25–35 Hz frequency band, located within aSTG (MNI coordinates −34,20,-32). This site was close to coordinates previously implicated in verbal semantic tasks (Binney, Embleton Karl, Jefferies, Parker, & Lambon Ralph, 2010). aSTG is expected to show a stronger response to taxonomic than thematic judgements according to the Dual Hub theory. In contrast, the Controlled Semantic Cognition account anticipates that both taxonomic and thematic relations are represented within ATL. We might anticipate greater activation when there is stronger conceptual overlap between two words, since ATL has been linked to conceptual combination (e.g., Bemis & Pylkkänen, 2013); moreover, the Controlled Semantic Cognition theory might anticipate that this effect would be observed both for words sharing more versus fewer physical features (even when behavioural performance is matched), and for stronger versus weaker thematic links (which necessarily differ in behavioural performance).

We also selected a site in pMTG (MNI coordinates −50,−46,−6) showing a strong oscillatory response at 5–15 Hz and 25–35 Hz from 200 ms after the onset of the second word. This pMTG site was close to a peak for semantic control in a meta-analysis of neuroimaging studies (Noonan et al., 2013). pMTG is predicted to show a stronger response to thematic than taxonomic judgements according to the Dual Hub view. In contrast, the Controlled Semantic Cognition view suggests that this site should show greater oscillatory power when participants are required to understand a conceptual link between words that are only weakly related, since in these trials, the pattern of semantic activation established by the first word is less relevant to the conceptual link between the items. Consequently, more semantic control may be required from the onset of the second word to shape ongoing retrieval to suit the task demands.

Two other POI locations are provided in supplementary analyses. AG did not show a significant response in our whole-brain beamforming analysis and therefore the selection of a POI in this region was not strongly motivated. However, given the contrasting predictions that this site should respond to thematic over taxonomic relations (from Dual Hub theory) and to strong over weak thematic associations (from the Controlled Semantic Cognition account), we placed a POI in AG at coordinates implicated in ‘automatic’ semantic processing by a recent meta-analysis (MNI coordinates −48,−68,28) (Humphreys & Lambon Ralph, 2015). There was also a local peak in the whole brain beamforming data in left IFG pars triangularis from 200-400 ms at 25–35 Hz (MNI coordinates −40,30,−8). This site fell within the IFG cluster implicated in semantic control by the Noonan et al. (2013) meta-analysis. Therefore, it might be expected to show a response for weak > strong thematic associations and for harder taxonomic trials contrasted with easier strong thematic trials. For IFG, we did not have contrasting predictions from different theoretical accounts; however, for completeness, this site is included in the supplementary materials.

We elected to examine left-hemisphere sites since (i) fMRI and patient studies reveal a greater contribution of the left hemisphere to semantic processing in general (Binder and Desai, 2011, Binder et al., 2009); and (ii) right motor cortex was expected to show irrelevant responses related to the preparation of button presses with the left hand, even though button presses were only required on catch trials which were excluded from the analysis. At each POI, we contrasted the oscillatory response to (i) taxonomic and weak thematic trials matched in terms of behavioural performance, (ii) taxonomic and strong thematic trials, which were easier and (iii) strong and weak thematic trials with differing control demands at a high resolution in time and frequency. The time-series of each POI was reconstructed epoch by epoch, for each subject, by means of separate beamformers (Huang et al., 2004). Time-frequency analyses were computed using Stockwell transforms (Stockwell et al., 1996) over a time window from −800 to 700 ms (to avoid edge effects) and a frequency range from 5-50 Hz. The Stockwell transform, implemented in the NAF software, uses a variable window length for the analysis which is automatically adapted along the frequency range according to the sample rate and the trial length (4th order Butterworth filters with automatic padding).

We computed generalized linear mixed models (GLMM) to compare time-frequency representations across conditions using PROC MIXED in SAS (SAS Institute Inc., North Carolina, US). Time–frequency plots of signal change were treated as two dimensional arrays of small time-frequency tiles, indexed in the model by three main effects, each of which is defined as a class variable: time, frequency and the interaction between time and frequency. Therefore, random effects were included in each GLMM to account for the fact that each participant's time–frequency plot is made up of multiple time-frequency tiles. We also controlled for time-frequency (or spatial) co-variance in the spectrogram by assuming the estimates of power followed a Gaussian distribution: consequently a Gaussian link function was used in the model. The time-frequency (spatial) variability was integrated into the model by specifying an exponential spatial correlation model for the model residuals (Littel, Stroup, Milliken, Wolfinger, & Schabenberger, 2007). The time-frequency (spatial) variability of the S-transform (loss of frequency resolution at higher frequencies and loss of temporal resolution at lower frequencies) was accounted for by splitting the data in three frequency bands (5–15 Hz; 15–40 Hz; 40–50 Hz) to make the spatial smoothing more appropriate.

Finally, the data were resampled at a frequency resolution of 2 Hz and time resolution of 25 ms, the smallest time and frequency bin consistent with model convergence. This time-frequency resolution proved optimal in other similar published studies (Klein et al., 2014, Urooj et al., 2014, Wheat et al., 2010). PROC MIXED constructs an approximate *t* test to examine the null hypothesis that the LS-Mean for signal change between conditions was equal zero in each time-frequency tile, and the procedure automatically controls for multiple comparisons (i.e., controlling experiment-wise type I error). This method has been used in multiple peer-reviewed papers (Klein et al., 2014, Urooj et al., 2014, Wheat et al., 2010).

The POI analyses were computed using evoked as opposed to total power, since inspection of time–frequency plots for the whole trial revealed relatively little response to the first word but a strong evoked response following the onset of the second word, corresponding to the phase of the task when participants were evaluating the semantic link between the two items (See Supplementary Figure S1). Since this experiment contained a mixture of different types of semantic relationships, and not all of the items were globally associated, semantic retrieval to the first item may have been muted, in comparison with other similar studies in which the two semantically related words were always associated (e.g., Teige et al., 2018). The time-frequency representations of power were normalized, separately for each participant, with respect to the mean power per frequency bin in a baseline period prior to the start of trials across the conditions entered in the analysis (−700 to −500 ms prior to the onset of the second word). This window length was also used in earlier studies (Klein et al., 2014, Wheat et al., 2010), since it provides a compromise between the minimum length sufficient to estimate power at the lowest frequency we report (i.e., 5 Hz) and the requirement to characterise the state of the brain immediately before the onset of each trial.

The statistical contours characterising condition differences encompass time-frequency tiles fulfilling both of the following criteria: a) the difference between conditions reached *p* < .05; and b) the response was significantly different from baseline in at least one of the two contributing conditions at *p* < .01. In addition, since our statistical models were corrected for multiple corrections at each site, but not across the four POIs and three task contrasts, we also applied a cluster-size correction designed to control the probability of false positives across twelve analyses. For different fill rates (i.e., the number of time-frequency tiles showing a significant difference across conditions), we estimated the probability of obtaining different numbers of contiguous tiles by chance, assuming that the tiles showing a significant difference were randomly distributed over time-frequency space. This simulation is shown in Supplementary Figure S2. The maximum fill rate was 7.5% of the tiles. At this fill rate, a cluster size of six tiles reaches a Bonferroni-adjusted significance level of *p* < .05. Consequently, only significant clusters containing six or more tiles are enclosed by the statistical contours.

The analyses were not pre-registered prior to the research being conducted.

## 3 Results

### 3.1 Behavioural results

**Fig. 2:**
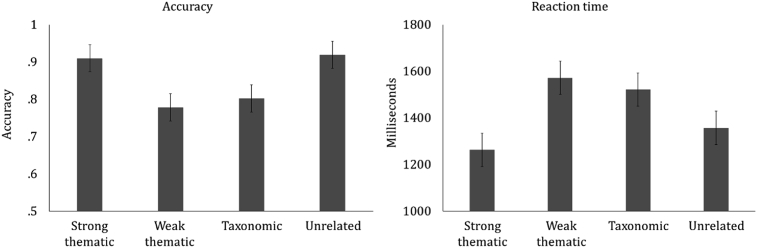
Accuracy and RT for catch-trial data in the taxonomic, strong thematic, weak thematic and unrelated conditions respectively. Error bars show SE.

The behavioural data from the catch-trials showed a significant difference in RT and accuracy between the strong and weak thematic conditions, while the taxonomic and weak thematic conditions were matched for behavioural performance (see Fig. 2 and Table 2).

**Table 2:** *t*-tests for RT and accuracy data from catch-trials collected during MEG recording.

Measure	Contrast	t	Sig (2-tailed)
Reaction time	Taxonomic/Thematic strong	2.37	**.03**
	Taxonomic/Thematic weak	−.57	.58
	Strong/Weak thematic	−3.10	**<.01**
Accuracy	Taxonomic/Thematic strong	−4.76	**<.001**
	Taxonomic/Thematic weak	1.00	.32
	Strong/Weak thematic	4.89	**<.001**

Footnote: Within-subjects comparisons. Degrees of freedom = 18. Significant effects are highlighted in bold text.

### 3.2 Whole brain beamforming

**Fig. 3:**
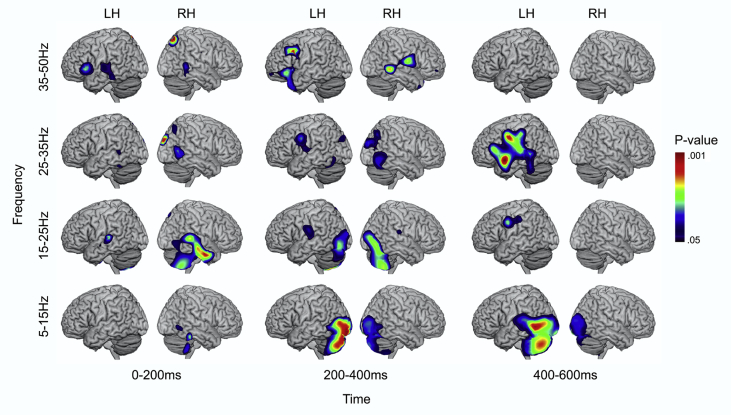
Whole brain beamforming data, comparing an active retrieval period, following the onset of the second word in the pair, with a passive period before the onset of the trial, across all semantically-related trials in four frequency bands. Map shows voxels in the brain with t-values equal or higher than the top 5% t-values present in the null distribution. Images created using MRIcron software (Rorden et al., 2007).

There were extensive changes in total oscillatory power, following onset of the second word in the pair, relative to the baseline period (see Fig. 3). These changes were maximal from 25-35 Hz and 400–600 ms post-target onset in regions within the semantic network. The earliest response to the task, from 0-200 ms, was seen in bilateral mid-STG and right ITG (from 15-25 Hz), left IFG (35–50 Hz) and secondary visual regions (across frequencies). In the subsequent period, 200–400 ms, there was a marked expansion of the visual response, particularly in the left hemisphere, extending into the cerebellum (from 5-25 Hz). There was also activation within left precentral gyrus (from 25-50 Hz) and insula, extending to ventral IFG and aSTG in the left hemisphere (from 35-50 Hz). From 400-600 ms, the visual response had spread to include left posterior temporal cortex (from 5-15 Hz) and there was also strong activation of semantic and language regions, including aSTG, IFG and pMTG, from 25-35 Hz.

### 3.3 POI results

For each site and contrast, we present evoked power across time and frequency for (i) taxonomic and strong thematic trials (TOP ROW), (ii) taxonomic and weak thematic trials (MIDDLE ROW) and (iii) strong versus weak thematic trials (BOTTOM ROW). The difference between these conditions is shown in the left-hand column. For the individual conditions, red-yellow colours indicate *increased* oscillatory power in the task relative to the passive period, while blue colours depict task-induced power *decreases* relative to this baseline. Regions of time-frequency shown in green are unchanged relative to the passive period. For the difference plots (i.e., normalized condition *A –* normalized condition *B*), red-yellow colours indicate regions where the power of condition A exceeds condition B (i.e., more power for taxonomic judgements – top/middle rows; or the strong thematic condition – bottom row), while blue colours indicate where the power in condition B exceeds condition A (i.e., more power for thematic judgements – top/middle rows; or the weak thematic condition – bottom row). The statistical contours indicated by solid black lines indicate regions fulfilling three criteria: i) there was a significant change from baseline in at least one of the conditions (at *p* < .01), (ii) the conditions were significantly different from each other (*p* < .05), (iii) the significant cluster included six or more contiguous tiles (see above for rationale). If criterion iii) was not met, the black line remains dashed.

#### 3.3.1 aSTG

**Fig. 4:**
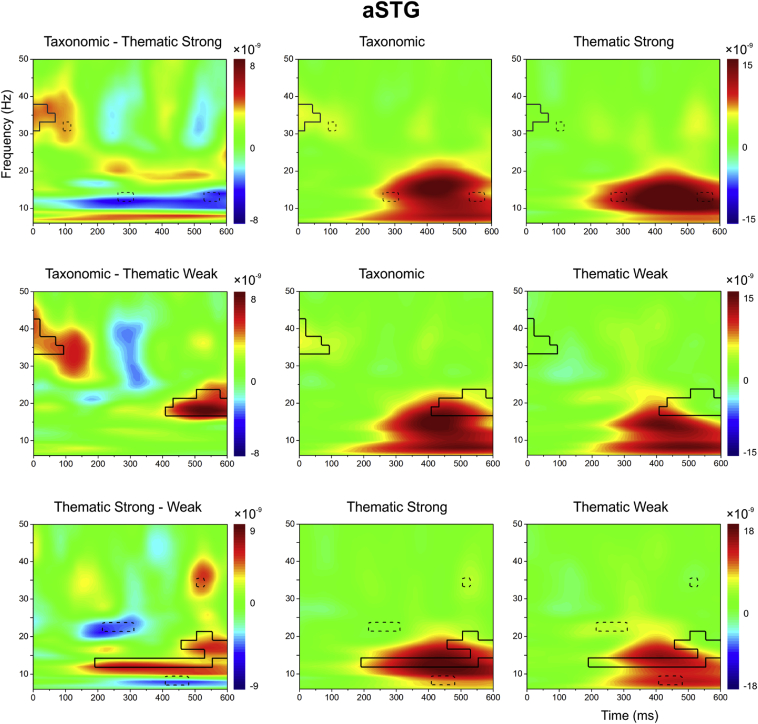
Evoked power in aSTG. Difference plots (left-hand column): Differences between taxonomic and strong thematic trials (TOP ROW), taxonomic and weak thematic trials (MIDDLE ROW) and strong versus weak thematic trials (BOTTOM ROW). The black lines mark statistical contours fulfilling the following criteria: a) the difference between conditions reached *p* < .05; b) the region was also significantly different from baseline in at least one of the two contributing conditions at *p* < .01; c) the cluster had six or more contiguous tiles showing a significant difference. The dotted lines show regions which fulfilled the first two criteria but had fewer than six tiles. The plots for each condition (middle and right-hand column) show signal change for each trial type relative to a “passive” baseline.

This site showed a strong evoked response to the presentation of the second word across conditions (see Fig. 4). This response commenced very rapidly following stimulus onset (during which time semantic processing established by the first item would have been ongoing) and was relatively broadband by 200 ms.

Statistical contrasts revealed a stronger response to taxonomic than both strong and weak thematic relations within the first 75 ms at 30–40 Hz, which could not be explained in terms of difficulty or semantic coherence as measured by word2vec. This response (although perhaps not its brief duration) is predicted by the Dual Hub account. In addition, the main broadband semantic response in aSTG showed an effect of semantic coherence across different tasks and contrasts. This site was sensitive to strength of association within thematic trials, with a stronger oscillatory response for strong versus weak thematic trials (i.e., when word2vec was higher). This difference commenced at 200 ms after the onset of the second word and lasted for the rest of the epoch, from 12-22 Hz. The effect of strength of association within thematic trials also overlapped with the contrast between taxonomic versus weak thematic trials. aSTG showed a stronger response to taxonomic trials (with higher word2vec scores), from 400-600 ms from 15-25 Hz. The observation that aSTG showed sensitivity at the same time and frequency to two contrasts in which word2vec differed – reflecting shared physical features (taxonomic *vs* weak thematic trials) and thematic co-occurrence between two successive words – is consistent with view that this site responds more to semantically coherent inputs, irrespective of the type of feature that drives this coherence. Differences in difficulty did not provide an adequate explanation for this pattern (as behavioural performance and rated difficulty was matched for the taxonomic *vs* weak thematic trials).

#### 3.3.2 pMTG

**Fig. 5:**
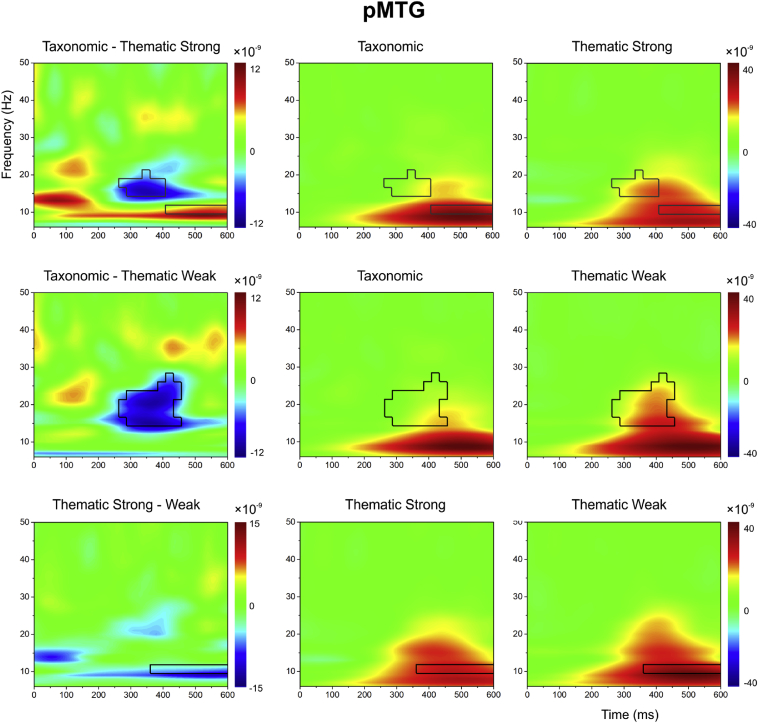
Evoked power in pMTG. Difference plots (left-hand column): Differences between taxonomic and strong thematic trials (TOP ROW), taxonomic and weak thematic trials (MIDDLE ROW) and strong versus weak thematic trials (BOTTOM ROW). The black lines mark statistical contours fulfilling the following criteria: a) the difference between conditions reached *p* < .05; b) the region was also significantly different from baseline in at least one of the two contributing conditions at *p* < .01; c) the cluster had six or more contiguous tiles showing a significant difference. The dotted lines show regions which fulfilled the first two criteria but had fewer than six tiles. The plots for each condition (middle and right-hand column) show signal change for each trial type relative to a “passive” baseline.

Like aSTG, this site showed a strong evoked response to the presentation of the second word, particularly between 5 and 30 Hz (see Fig. 5). The response was apparent 100 ms after the onset of the second word and was relatively broadband by 250 ms. Statistical contrasts between taxonomic and thematic trials revealed a stronger response to thematic than taxonomic relations from around 250-400 ms, between 15 and 25 Hz. This effect was observed for both weak and strong thematic trials (i.e., even when the thematic task was easier than the taxonomic task), consistent with the predictions of the Dual Hub account, which proposes that pMTG (along with AG) plays a critical role in representing semantic relationships and events.

Unambiguous effects of control demands were also observed in pMTG, irrespective of the type of semantic judgement. There was a strong oscillatory response to weak versus strong thematic associations at a relatively late time-point (400–600 ms; around 8 Hz). This effect of strength of association in pMTG is consistent with fMRI studies, which have repeatedly observed greater activation in left pMTG (alongside IFG) for weak relative to strong associations that require more controlled retrieval (Davey et al., 2016, Hallam et al., 2016, Noonan et al., 2013). An overlapping response to more difficult trials was found for the taxonomic > strong thematic contrast, indicating that the contribution of pMTG to semantic control is not restricted to thematic relations. Moreover, left pMTG and IFG are thought to be key regions in a network supporting semantic control (Davey et al., 2016, Noonan et al., 2013) and left IFG also showed a stronger oscillatory response in the contrast of taxonomic versus strong thematic relations, at the same frequency, but earlier in time (from 100-350 ms; see Supplementary Figure S4).

#### 3.3.3 Summary of results

The two temporal lobe sites showed opposite effects of difficulty (irrespective of the type of relation). Also, at different times and frequency bands, they showed opposite effects in contrasts between thematic and taxonomic relations (irrespective of difficulty). aSTG showed a larger oscillatory response for taxonomic relative to thematic judgements, and for strong versus weak thematic judgements, while pMTG showed the contrary pattern (thematic > taxonomic and harder > easier trials). The main response to the task within aSTG was stronger for pairs of items that were more semantically coherent (according to word2vec scores) – in other words, where the pattern of semantic retrieval elicited by the first word was more relevant to the conceptual link between the two words that participants were required to retrieve. This effect could be driven by overlapping physical features (in the contrast of taxonomic *vs* weak thematic trials) and item co-occurrence (in the contrast of strong *vs* weak thematic trials), in line with predictions for a semantic store that is sensitive to both types of conceptual relationship (as anticipated by the CSC framework). There was also an early gamma band response to taxonomic relations that could *not* be explained in terms of overall semantic coherence: this finding taken in isolation is consistent with the Dual Hub account, although this framework might anticipate that this effect would last for a longer duration, and modulate the main task response.

In contrast, pMTG showed a stronger response when participants were asked to identify the connection between words that lacked a strong thematic link. This site showed a sensitivity to strength of association (weak > strong thematic relations) and to task demands (taxonomic > strong thematic relations). These effects of difficulty were highly overlapping in time and frequency (occurring in the alpha band at 10 Hz), consistent with a role for pMTG in semantic control across tasks. Stronger oscillatory power in pMTG was also seen for thematic versus taxonomic decisions in the beta band, across both strong and weak thematic conditions. This suggests there is a separate response in pMTG, which is stronger for thematic than taxonomic decisions, and which can be observed even when the thematic task is easier.

## 4 Discussion

In a two-word association judgement task, we compared the oscillatory response across nodes of the semantic network (i) for taxonomically and thematically-associated word pairs (i.e., mole-cat*vs*cushion-cat), and (ii) for easier and harder thematic pairs with different levels of associative strength (i.e., strongly-linked items such as milk-cat*vs* weakly-linked items such as cushion-cat). We contrasted the predictions of the Dual Hub theory (which anticipates a dissociation between semantic sites by type of judgement) and the Controlled Semantic Cognition account (which anticipates a dissociation according to semantic control demands). Dual Hub theory predicts that ATL is important for taxonomic relationships while left pMTG and/or AG support thematic knowledge (Schwartz et al., 2011). Alternatively, the Controlled Semantic Cognition account postulates a single representational hub (ATL) underpinning knowledge of all types of relationship (Hoffman et al., 2018, Jackson et al., 2015, Lambon Ralph et al., 2017). When the task requires activation within the semantic store to be shaped to suit the demands of the task or the context, as for weak associations, this framework predicts greater engagement of sites implicated in ‘semantic control’ – namely left IFG and pMTG (Jefferies, 2013, Noonan et al., 2013, Whitney et al., 2011).

We observed functional dissociations relating to *both* the type of conceptual relationship (taxonomic *vs* thematic) and semantic control demands within the left temporal lobe. The response in aSTG (within ATL) was stronger for (i) *taxonomic* judgements, relative to thematic judgements, as well as for (ii) *strong* versus weak thematic judgements. In complete contrast, pMTG showed a stronger oscillatory response for (i) *thematic* versus taxonomic judgements and for (ii) *weak* versus strong thematic judgements. Many although not all of our findings can be accounted for by the suggestion that ATL is sensitive to the *semantic coherence* between successive concepts, while pMTG supports *controlled semantic* processes, which shape the dynamic pattern of retrieval that follows the first word to identify semantic overlap with the second word. We can define semantic coherence as the extent to which the meanings of two items are consistent with each other – with word2vec ratings providing a numeric estimate of this variable. Since we have knowledge of both the physical features of concepts and their associations, coherence is relevant for both taxonomically-linked and thematically-linked pairs. Strongly-associated words generate more coherent patterns of activation than weakly-associated words because the past co-occurrence of these concepts will have strengthened the link between them; moreover, taxonomic pairs, which share many physical features, are more semantically coherent (and have higher word2vec scores) than difficulty-matched thematic pairs, which share few features. By this view, ATL is not only sensitive to taxonomic relations, but its response is modulated more generally by the degree to which items generate coherent patterns of semantic activation. Moreover, when coherence is low and the first word does not establish a pattern of semantic retrieval consistent with the link to be retrieved, there is a strong requirement to shape ongoing retrieval, recruiting pMTG. This proposal potentially explains both contrasts of strong versus weak thematic and taxonomic versus weak thematic conditions at both sites. However, it does not provide an explanation for the early response to taxonomic overlap in ATL, or the separate responses seen in pMTG to difficulty and thematic decisions. Below, we discuss the contributions of each site to semantic cognition, and seek to explain findings consistent with both the Dual Hub and Controlled Semantic Cognition frameworks.

Our results, taken together, are inconsistent with the view that difficulty, as measured by behavioural performance, is sufficient to explain functional dissociations within the semantic system: Jackson et al. (2015) found no differences between taxonomic and thematic judgements when statistically controlling for response time, and suggested that previous studies identifying different neural substrates for taxonomic and thematic decisions might be explained by the confounding effect of difficulty. However, the sensitivity of MEG to effects through time and frequency revealed a dissociation between taxonomic and thematic trials, irrespective of difficulty (and even when there were independent effects of difficulty at distinct points in time-frequency in the same contrast). Our results also show that multiple factors contribute to the difficulty of semantic decisions. Semantic overlap (as assessed by word2vec) is expected to make semantic decisions easier, because the first item sets up more task-relevant patterns of semantic activation. However, taxonomic trials are harder than thematic trials with comparable semantic overlap, as assessed by behavioural performance and participants' ratings – perhaps because participants tend to think in terms of thematic relations when given a free choice (Lin & Murphy, 2001).

### 4.1 Anterior superior temporal gyrus within ATL

In this study, aSTG showed a stronger evoked response for taxonomic than thematic judgements, but this site was also sensitive to strength of association. The Dual Hub theory suggests that ATL contributes to taxonomic aspects of conceptual knowledge (Schwartz et al., 2011); for example, patients with lesions in this region are reported to make more taxonomic than thematic errors in picture naming. However, the Controlled Semantic Cognition framework emphasises the way in which ATL integrates a wide-range of features – including sounds, motor features, linguistic information, spatial/episodic representations and valence (Lambon Ralph et al., 2017). The convergence of these inputs is thought to be graded within ATL: there is a larger response to words in aSTG and to pictures in anterior fusiform, while middle and inferior temporal gyrus show a heteromodal response consistent with integration of both verbal and non-verbal features (Murphy et al., 2017, Visser and Lambon Ralph, 2011). This theoretical framework can account for multiple aspects of our data. First, the peak response across conditions in the whole brain beamforming analysis fell within aSTG, which likely reflects the verbal nature of the semantic task we used (Hoffman, Binney, & Lambon Ralph, 2015). Secondly, the diversity of information integrated within ATL might allow multiple types of semantic relations to be computed across this region (both taxonomic and thematic). The items in taxonomic pairs by definition shared a wider range of features than those in weak thematic pairs and we propose this coherence between features gave rise to the stronger ATL response in this condition.

Our findings replicate the strong > weak effect we observed within ATL in a recent MEG study (Teige et al., 2018) as well as a recent fMRI study in which aSTG increased its response to verbal semantic judgements presented in a supportive semantic context (a relevant preceding sentence; Hoffman et al., 2015). More broadly, the larger response to both strong associations and taxonomic pairs sharing many features is consistent with the proposal that ATL is sensitive to coherent conceptual combinations (Davey et al., 2016, Poortman and Pylkkänen, 2016). For example, MEG studies have shown a stronger response in ATL for word pairs that can be combined in a meaningful way, such as red and boat, compared with cup and boat (Bemis & Pylkkänen, 2013). We speculate that when patterns of semantic retrieval required by a task are highly consistent with the most accessible conceptual information in ATL (either features primed within a taxonomic decision or strong associations for a thematic decision), the response of this region is increased since coherent patterns of semantic retrieval are stable and self-reinforcing (McClelland & Rogers, 2003). In these circumstances, the role of additional systems that can constrain semantic retrieval to suit the circumstances may be minimised (see below). This proposal is consistent with the observation that the main oscillatory response in aSTG was modulated, in overlapping parts of time-frequency space, by both the contrast of strong > weak thematic associations and the contrast of taxonomic > weak thematic trials (with higher word2vec scores in the taxonomic condition, yet matched for behavioural performance).

MEG is able to characterise the neural response as semantic retrieval emerges over time. In line with our data, the existing literature characterises semantic retrieval as having both early and late components. The peak response was around 400 ms post stimulus onset, which corresponds to the N400 (Vartiainen, Parviainen, & Salmelin, 2009). This effect has been localised to anterior-to-mid temporal cortex (Lau et al., 2014). However, effects of semantic factors can occur much more rapidly than this (Bemis and Pylkkänen, 2013, Chan et al., 2011, Clarke et al., 2011, Marinkovic et al., 2003, Mollo et al., 2017). The current study revealed two phases of response in aSTG. First, there was a very early response within 100 ms of stimulus onset, at 40 Hz, which was stronger for taxonomic trials contrasted both with weak and strong thematic relations, irrespective of difficulty. This result is consistent with an emerging literature that suggests that rapid recurrent activation between visual, semantic and articulatory codes occurs during reading, as opposed to semantic access emerging as a final stage of processing (Klein et al., 2014, Wheat et al., 2010, Yvert et al., 2012). This is also in line with the emerging view that gross categorical information can be extracted for written words within 100 ms (Chan et al., 2011, Mollo et al., 2017). Since this effect was present for taxonomic relations and not thematic relations, irrespective of strength of association, overlap between physical features may be identified more quickly within ATL than strong thematic relationships. In our paradigm, participants did not know which type of relationship was going to be probed on a given trial, and so this initial response in ATL following the onset of the second word may have enabled the semantic network to be configured appropriately for subsequent conceptual processing. The absence of any overlap between gross categorical information for the second word and semantic features previously activated by the first word would suggest the need to identify a thematic context linking the words.

There was also a later phase of the response in aSTG, from 400 ms onwards at 20 Hz. This was the peak oscillatory response across conditions, which showed both strong > weak thematic and taxonomic > weak thematic effects. However, there was no difference in this phase between conditions matched for word2vec scores (taxonomic = strong thematic trials). It is possible to account for these overlapping effects of strength of association and type of semantic relation in aSTG in terms of a single neurocognitive effect – semantic coherence – common to both contrasts (although the strong > weak thematic effect emerged earlier, by 200 ms). Both of these differences reflected a more sustained oscillatory response when the two words had a greater overlap in their meanings (as indexed by word2vec) consistent with the hypothesis above that semantic coherence in ATL gives rise to a more stable and self-reinforcing pattern of retrieval, irrespective of whether this overlap relates to shared physical features or a frequently-occurring common context.

### 4.2 Posterior middle temporal gyrus

pMTG is implicated in diverse aspects of semantic cognition. By one view, it acts as an interface between lexical and conceptual representations, allowing semantic access from language – although fMRI studies show a multimodal response to both verbal and non-verbal semantic tasks (Krieger-Redwood et al., 2015, Vandenberghe et al., 1996, Visser et al., 2012). Second, pMTG is associated with understanding thematic relations, events and actions (Gennari et al., 2007, Kable et al., 2005, Martin and Chao, 2001, Perani et al., 1999, Shapiro et al., 2005, Tranel et al., 2003, Vigliocco et al., 2011). A third set of studies show that pMTG participates in a left-lateralised network underpinning semantic control, along with anterior IFG (Davey et al., 2016, Hallam et al., 2016, Jefferies, 2013, Noonan et al., 2013, Teige et al., 2018, Wang et al., 2018, Whitney et al., 2011). We observed a greater engagement of pMTG for thematic than taxonomic relationships (irrespective of difficulty), and for weak compared with strong thematic relationships. Our results are therefore consistent with the possibility pMTG supports both knowledge of thematic relations and semantic control processes, in line with previous observations using fMRI (Davey, Cornelissen et al., 2015, Davey, Rueschemeyer et al., 2015).

There are several ways that these findings might be explained. One possibility, anticipated by the Controlled Semantic Cognition framework, is that a ‘hub and spoke’ architecture for semantic representation interacts with semantic control processes. By this view, pMTG might be a ‘spoke’ representing action features or multimodal aspects of event knowledge, as well as a key region in the network underpinning semantic control. Since MEG lacks the spatial resolution to separate proximal sources, there could be distinct regions of pMTG associated with processing thematic relations (irrespective of difficulty) and semantic control. This possibility is consistent with our observation that the effects of thematic judgements and difficulty were non-overlapping in time-frequency. The thematic > taxonomic contrast occurred within the beta band, which has been associated with the retrieval of action semantics and syntactic binding processes – aspects of cognition which may relate to thematic processing (for a review, see Weiss & Mueller, 2012). In contrast, the effect of difficulty across taxonomic and thematic judgements occurred within the alpha band, which has been linked to controlled access to semantic information and to sustained patterns of focussed retrieval (for a review, see Klimesch, 2012).

These effects of thematic processing and semantic control might be fully independent and driven by distinct sites within pMTG. Alternatively, there might be shared computational principles which relate to both the effects of semantic control and semantic relation (thematic > taxonomic), given that action/event understanding and semantic control recruit similar neural networks in functional neuroimaging studies (Davey, Rueschemeyer et al., 2015, Mollo et al., 2018). We previously suggested that pMTG might support the dynamic updating of a conceptual ‘context’, corresponding to aspects of semantic information which are currently relevant (Teige et al., 2018). This context could bias retrieval within the long-term semantic store, allowing adaptive semantic cognition. Both thematic relations *and* controlled retrieval might be supported by this type of mechanism, since in both situations, there is a requirement to vary retrieval over time, according to the circumstances. For thematic trials, participants must generate a spatiotemporal context in which concepts co-occur (e.g., for dog-brush, a context such as grooming). This conceptual context can then promote relevant features and associations (e.g., fur). In contrast, the items in taxonomic trials do not co-occur within a specific spatiotemporal context, potentially reducing recruitment of pMTG at from 300-400 ms in the beta band; instead shared physical features – potentially detected at a very early time-point in ATL – might provide evidence of a link between the concepts (see Thompson et al., 2017, for a related argument).

Similarly, in tasks that engage semantic control processes, specific non-dominant features or associations need to be prioritised over more strongly encoded but irrelevant aspects of knowledge – and again the subset of knowledge that must be selected in line with the current task varies over time. Semantic selection processes are likely to be engaged more strongly for weak thematic trials, because for these items, the dominant association to the first item elicits features which are inconsistent with the second item. For example, in a trial such as police-lamp, ongoing semantic retrieval from the first word (handcuffs; criminal) is not highly related to the meaning of the second item (light), and therefore, to determine these words are in fact related, it is necessary to (i) identify a linking context (from 250-400 ms in the beta band) and (ii) use this linking context to selectively focus retrieval on features relevant to the conceptual overlap between the two words (e.g., bluelight and darknight, from 400 ms in the alpha band). Although weak thematic associations had the highest control demands in this study, the taxonomic trials in our experiment also required participants to selectively focus on shared physical features of the probe and target (e.g., lamp and sun are both bright), as opposed to dominant associations, such as the fact that lamps are found on desks). Therefore semantic selection processes from 400 ms in the alpha band are not restricted to thematic trials. In line with this proposal, a recent study found that the degree of feature overlap between the items in taxonomic judgements modulated pupil size (as a marker of cognitive control), even more than strength of association for thematic judgements (Geller, Landrigan, & Mirman, 2019).

MEG provides unique information about the time-course of semantic control processes that shape semantic retrieval to suit the context. Our data suggests the identification of a linking context in thematic trials from 250–400 ms immediately precedes semantic control processes recruited in both taxonomic and weak thematic trials (from 400 ms onwards). This pattern is consistent with the suggestion that the activation of a linking context can then guide the selection of semantic information in pMTG. However, this account remains highly speculative and in need of further investigation.

### 4.3 Broader networks encompassing aSTG and pMTG

**Fig. 6:**
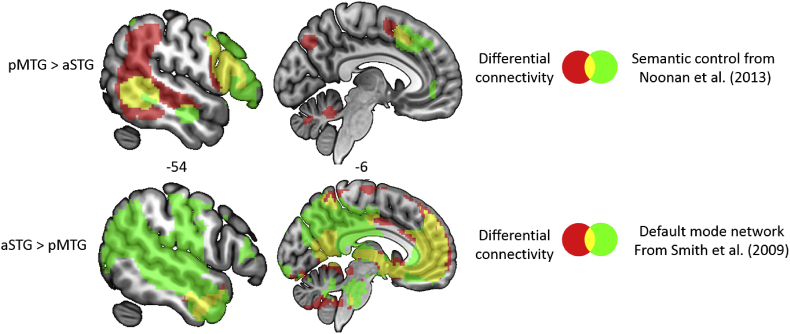
Patterns of intrinsic connectivity for the two VE sites within the temporal lobe, demonstrating overlap with distinct large-scale networks.

The two temporal lobe sites sensitive to semantic coherence (aSTG) and contextually-guided retrieval (pMTG) respectively fell within distinct large scale networks. To aid interpretation of the MEG findings, we characterised these networks using measures of intrinsic connectivity measured by fMRI at rest (see Fig. 6). When the connectivity patterns of the two sites were contrasted, aSTG showed greater connectivity to sites within the default mode network (DMN), particularly posterior cingulate cortex and medial prefrontal cortex (see Davey et al., 2016, Binder et al., 2009, for related observations). Other regions within DMN, most notably AG, have also been implicated in coherent conceptual combinations (Bemis and Pylkkänen, 2013, Davey, Cornelissen et al., 2015). There was no significant response to semantic retrieval within AG in our whole brain beamforming analysis, and consequently we lacked a rationale for placement of a VE at this site. However, given that AG is associated with thematic as opposed to taxonomic knowledge (Schwartz et al., 2011), as well as automatic as opposed to controlled semantic processing (Davey, Cornelissen et al., 2015, Humphreys and Lambon Ralph, 2015), we placed an POI at the peak coordinates for “automatic semantics” in a recent meta-analysis (see Supplementary Materials). At this AG site, we observed greater oscillatory power for strong than weak associations, and no difference between taxonomic and thematic judgements matched for difficulty, consistent with the suggestion that AG might also contribute to semantic retrieval when inputs are highly coherent and mutually-reinforcing. When taxonomic and thematic strong trials matched on word2vec were compared, the magnitude of the overall oscillatory response was similar, but the taxonomic relations elicited a stronger response at an earlier time-point and lower frequency. These results demonstrate a functional dissociation between AG and pMTG in the effect of strength of association (along with other evidence; Davey et al., 2016, Humphreys and Lambon Ralph, 2015, Davey, Rueschemeyer et al., 2015); while pMTG is implicated in thematic processing (in line with the version of the Dual hub theory advocated by Mirman et al., 2017), AG shows a different response profile.

pMTG showed stronger intrinsic coupling to brain areas implicated in semantic control – namely IFG and pre-supplementary motor area; regions that showed reliable activation across different contrasts tapping semantic control in the meta-analysis of Noonan et al. (2013; see Fig. 6). Since left IFG is associated with semantic control across studies to a greater extent than pMTG (for example, in the meta-analysis of Noonan et al., 2013), we might expect this region to show stronger oscillatory power to weak than strong associations. No clear differences emerged in the contrast of weak > strong thematic relations and when hard taxonomic trials were contrasted with easier strong thematic relations, there were effects in both directions (see Supplementary Materials). These unexpected results might reflect the complex relationship between BOLD responses in fMRI studies and MEG measurements of oscillatory power: while the contribution of LIFG is clearly demonstrated in fMRI, the role of pMTG may be more prominent in MEG (see also Teige et al., 2018, for similar results).

### 4.4 Limitations

Our analysis examined oscillatory dynamics from the onset of the second word within a pair, when semantic retrieval was already underway; consequently timings are unlikely to be comparable to studies presenting single items. Moreover, we used whole-brain beamforming to localise peak responses within regions-of-interest identified from the fMRI literature. This approach cannot uncover a role for other sites, and MEG is likely to lack the spatial resolution needed to examine functional dissociations within ATL and posterior temporal cortex. In addition, while we have characterised the oscillatory dynamics underpinning semantic retrieval in different circumstances, we lack an overarching explanation of the functional significance of oscillations at specific frequencies. It has been argued that low and high frequency oscillations may reflect different underlying processes, with high frequency oscillations (>30 Hz) reflecting local interactions within a neural population, and low frequency oscillations (<30 Hz) underpinning coordination of distributed neural populations (Donner & Siegel, 2011). This is potentially consistent with the observation that early taxonomic effects in aSTG were at a relatively high frequency, while later effects of semantic coherence were at a lower frequency, but more research is needed to understand these differences.

### 4.5 Conclusion

While aSTG and pMTG showed a stronger response to taxonomic and thematic semantic decisions respectively, in line with the version of the Dual Hub theory proposed by Mirman et al. (2017), other aspects of our data suggest a dissociation between these sites in terms of coherent versus contextually-guided retrieval. aSTG showed sensitivity to the strength of association within thematic trials – i.e., a larger response when the items were strongly associated; a pattern which might not be expected for a ‘taxonomic hub’. pMTG showed clear-cut effects of strength of association in the opposite direction – i.e., a stronger oscillatory response when controlled retrieval demands were higher. These effects are consistent with the view that aSTG is sensitive to the *coherence* of both concrete features and thematic links, while pMTG shows stronger recruitment when it is necessary to identify a linking context, and/or to focus retrieval on specific aspects of knowledge.

## Open practices

The study in this article earned an Open Materials badge for transparent practices. Materials for the study are available on the Open Science Framework (osf.io/mz52c) and analysis code is available at vcs.ynic.york.ac.uk/naf/naf.

## CRediT authorship contribution statement

**Catarina Teige:** Conceptualization, Data curation, Formal analysis, Writing - original draft. **Piers L. Cornelissen:** Conceptualization, Methodology, Supervision, Visualization, Writing - review & editing. **Giovanna Mollo:** Supervision, Methodology. **Tirso Rene del Jesus Gonzalez Alam:** Formal analysis. **Kristofor McCarty:** Methodology. **Jonathan Smallwood:** Conceptualization, Writing - review & editing. **Elizabeth Jefferies:** Conceptualization, Project administration, Writing - review & editing.
