# Supplementary material for: Dissociations in semantic cognition: Oscillatory evidence for opposing effects of semantic control and type of semantic relation in anterior and posterior temporal cortex
Source: Cortex. 2019 Nov;120:308–25. doi: 10.1016/j.cortex.2019.07.002 (PMC6854548; doi:10.1016/j.cortex.2019.07.002)
Supplement: Multimedia component 1 [file mmc1.docx]

**Supplementary materials**

Supplementary Analysis 1: Whole epoch data

Our main analyses examined differential responses to the *second* word in each pair, since the second item had varying links with the first word across the taxonomic, thematic strong and thematic weak trials. In these supplementary analyses, we characterise the response to the whole epoch (i.e., to both words), to facilitate comparisons with other studies. Supplementary Figure 1 shows evoked and total power across both words in the pair, relative to a passive window prior to the onset of the trials (see Methods). Yellow-red colours indicate *power increases*, whereas cyan-blue colours indicate *power decreases* relative to the passive baseline period. Green indicates no change from baseline. The white lines indicate the presentation duration for the first word, while the black lines show this for the second word.

We compared the current experiment with a previous study from our group (Teige et al., 2018), which also presented pairs of words, using the same timings as in the current study, and which used a similar analysis pipeline for sites in ATL and pMTG. However, there was an important difference between these tasks: here, participants were presented with a range of different types of semantic relationship and not all words were globally related – this limited the utility of retrieving semantic associations from the first word. In contrast, in our previous study, all of the words were globally related and therefore participants would have been encouraged to retrieve related items from the first item, in anticipation of the second item.

There was a striking difference in the oscillatory response across these experiments, demonstrating how subtle task differences that affect the cognitive state of participants might change the structure of the oscillatory response. In the current study, there was a large evoked response to the second but not the first word, at both temporal lobe sites. The response in total power, relative to the passive period before the onset of the trial, was largely in a positive direction and sustained across the epoch, with this signal strengthening after the presentation of the second relative to the first item. In contrast, our previous study employing globally-associated pairs showed a strong evoked response to the first word, which was muted by the second word. The response to the second word was characterised by a strong reduction in total oscillatory power relative to the passive window, starting at or around the offset of the first word, which might reflect an increase in desynchronised neuronal activity (Hanslmayr, Staudigl, & Fellner, 2012). This comparison suggests that task requirements can have a large effect on the neuromagnetic response, even when stimuli are presented in a near-identical similar manner.

Across the two experiments, we replicated the effects of strong>weak in ATL and weak>strong in pMTG, even though these studies employed different participants and presented different sets of words (see Teige et al., 2018, for effects of strength of association in the earlier study). However, the timing of semantic effects in these two sites was reversed. In the current study, when participants did not know in advance how the two items would be related, the earliest response was within ATL: the match between semantic features activated in response to the first word and the gross catgeorical information available early in ATL for the second word (Chan et al., 2011) might have established the nature of the semantic link. In contrast, in our previous study when participants knew that items were globally associated (Teige et al., 2018), they were more likely to recover associations from the first item and maintain this context to shape semantic retrieval to the second word. Under these circumstances, pMTG showed a very early response to weak > strong associations, consistent with the view that this site was maintaining dominant semantic contexts and responded more strongly when the second item violated these expectations.


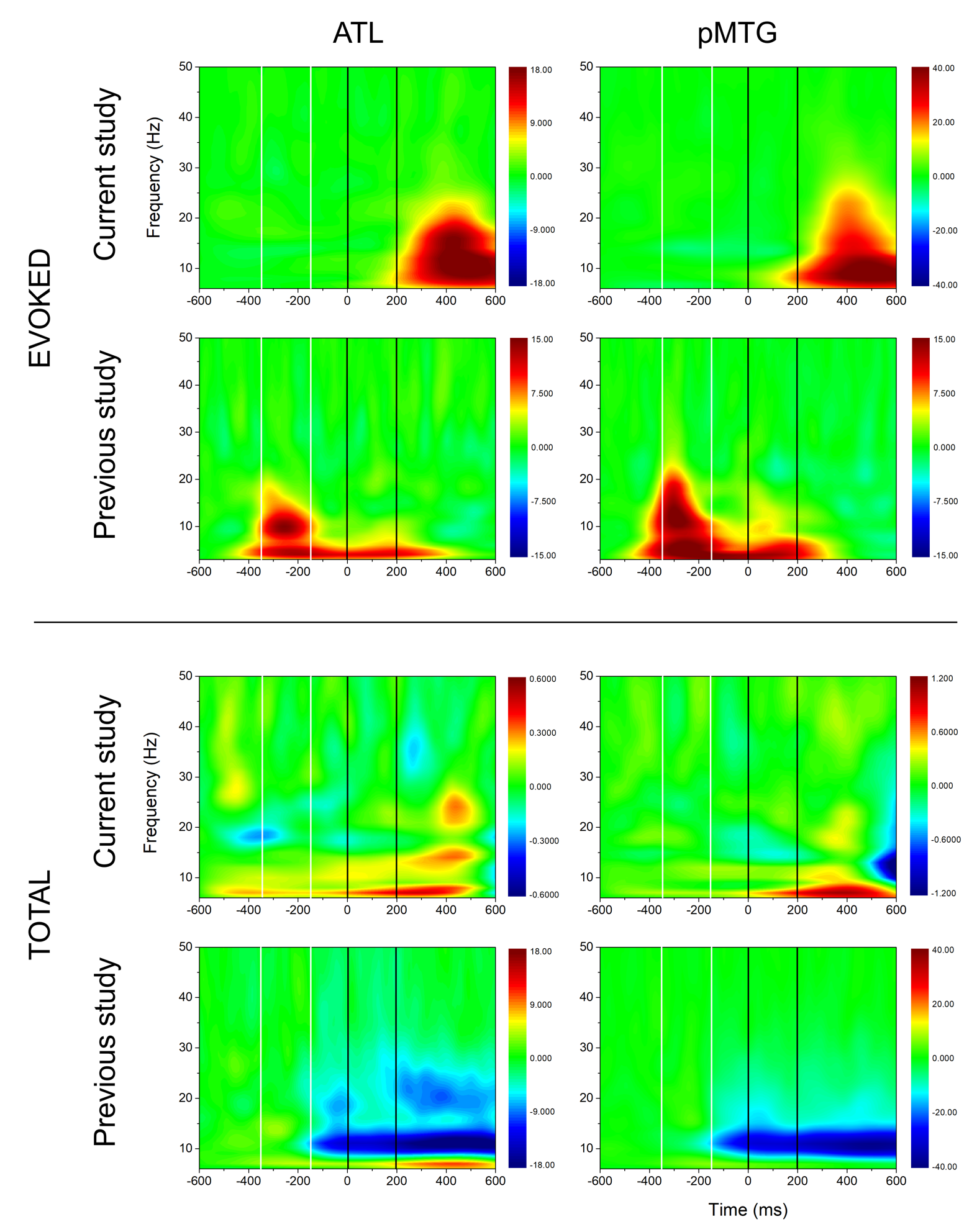


**Supplementary Figure S1**: TOP PANEL: Evoked power (i.e., phase-locked). BOTTOM PANEL: Total power (i.e., phase-locked and non-phase locked) for two sites (ATL & pMTG) across two experiments with identical presentation timings but different task requirements. Plots show the whole epoch (-400ms to 600ms) for related trials only. Presentation of the first word of the pair is shown with white vertical lines, while the second word is denoted with black vertical lines. The 0ms point marks the onset of the second word (since this was the primary analysis in both studies). The plots show signal change relative to a passive baseline 500-700 ms prior to target onset.

Supplementary Analysis 2: Computing a statistical threshold based on cluster size

Our statistical models for examining condition differences in time-frequency data were corrected for multiple corrections at each site, but not across the four POIs and three task contrasts. Therefore, we also applied a cluster-size correction designed to control the probability of false positives. For different fill rates (i.e., the number of time-frequency tiles showing a significant difference across conditions), we estimated the probability of obtaining different numbers of contiguous tiles by chance, assuming that the tiles showing a significant difference were randomly distributed over time-frequency space. This simulation is shown below in Supplementary Figure S2. Assuming a fill rate of no more than 8% of the time-frequency distribution, a cluster size of six or more tiles reaches a Bonferroni adjusted p value of 0.05 (i.e., a p value of 0.004, to account for twelve contrasts). Consequently, only significant clusters containing six or more tiles are enclosed by the statistical contours.


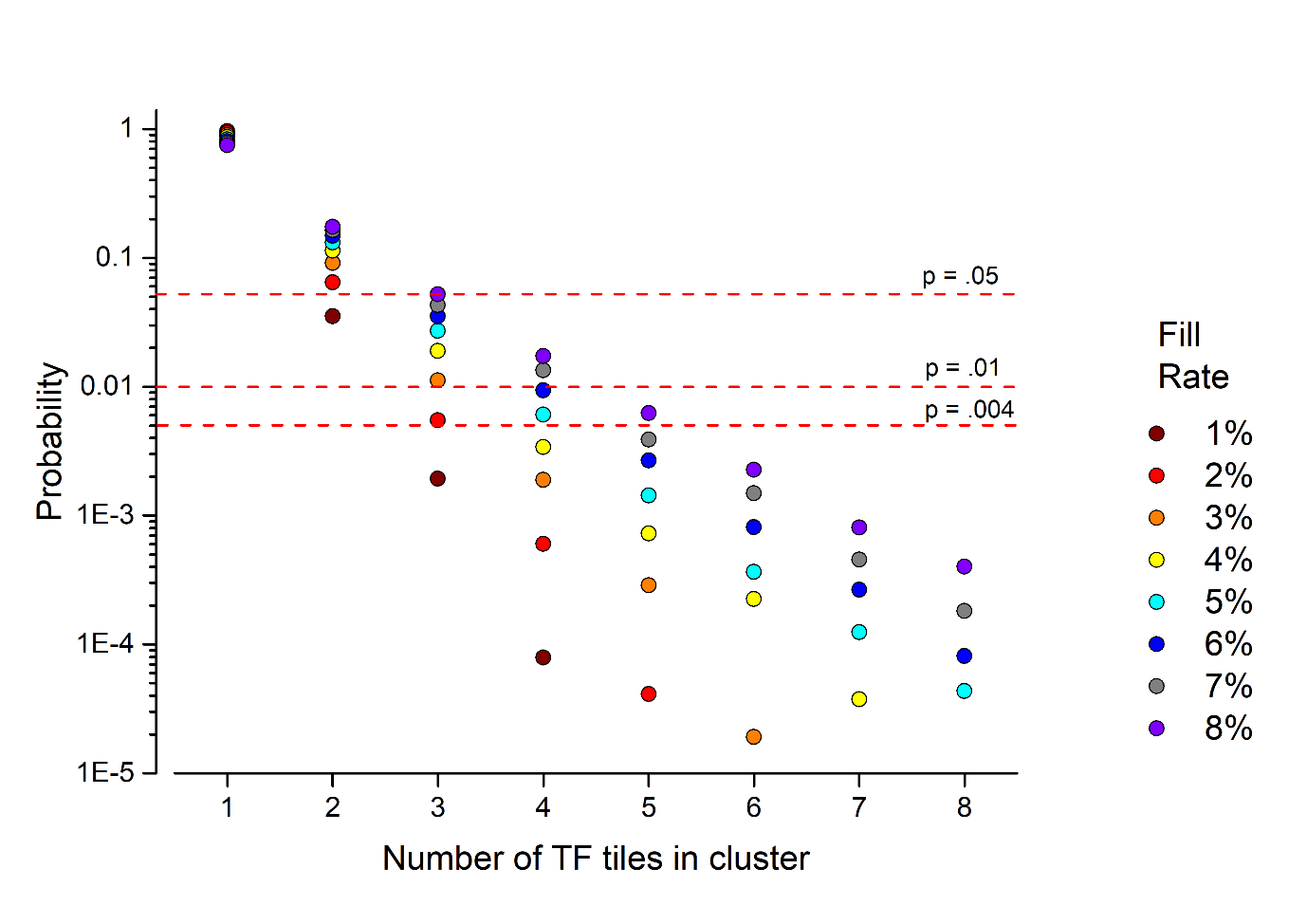


**Supplementary Figure S2**: The probability of observing clusters containing different numbers of contiguous tiles in the TF (time-frequency) plots, given different fill rates.

Supplementary Analysis 3: Time-frequency analyses of other semantic sites

We focussed our main analysis on sites which showed strong activation to the task across conditions, and for which the Dual Hub and Controlled Semantic Cognition framework made alternative predictions. However, for completeness, we present time-frequency analyses for two additional sites implicated in semantic cognition, angular gyrus (AG) and inferior frontal gyrus (IFG).

Angular Gyrus: AG did not show significant changes from baseline during the task in our whole-brain beamforming analysis, and so we lacked a strong rationale for examining this site. However, it is a purported thematic hub according to the Dual Hub account. As this site lies within the Default Mode Network, it might also show a stronger response to easy vs. difficult decisions.

Results: Time-frequency analyses are shown in Figure S3. AG showed no difference between thematic and taxonomic trials when these were matched for difficulty (taxonomic vs. weak thematic contrast). However, AG did show a stronger evoked response to strong vs. weak thematic associations, at 25-30Hz and from 350-450ms, consistent with the view that this site responds to more “automatic” patterns of semantic retrieval (Humphreys & Lambon Ralph, 2015). When easier strong thematic trials were contrasted with harder taxonomic trials, differences in both directions were observed. Easier thematic trials generated a stronger oscillatory response from 300-450ms at 15Hz (coinciding with the effect of difficulty in the thematic high vs. low contrast). Harder taxonomic trials also elicited an earlier low frequency response than strong thematic trials (100-250ms at 5-10Hz), but the interpretation of this effect is unclear given there was no comparable difference in the contrast of taxonomic with weak thematic trials.

Discussion: In fMRI studies, AG typically shows task-related deactivation relative to rest (Humphreys & Lambon Ralph, 2015), consistent with the fact that this site falls within the DMN. However, this site showed consistent activation for “automatic semantics” in a recent meta-analysis (Humphreys & Lambon Ralph, 2015), consistent with the high > low thematic association effect we observed. This result also aligns with a recent TMS study (Davey et al., 2015), which found that inhibitory stimulation of AG disrupted the efficient retrieval of strong associations, with stimulation of neighbouring pMTG producing the reverse pattern (greater disruption of weak association trials). Although strong associations are easier to retrieve than weak associations, there is some evidence that AG can show above baseline activation for even demanding semantic tasks that require attention to memory representations as opposed to the external world (Murphy et al., 2017). Moreover, like ATL, AG shows sensitivity to coherent conceptual combinations (Bemis & Pylkkänen, 2013), consistent with the view that these sites form a distributed functional network. Further research is needed to understand the complex pattern of differences observed at this site.


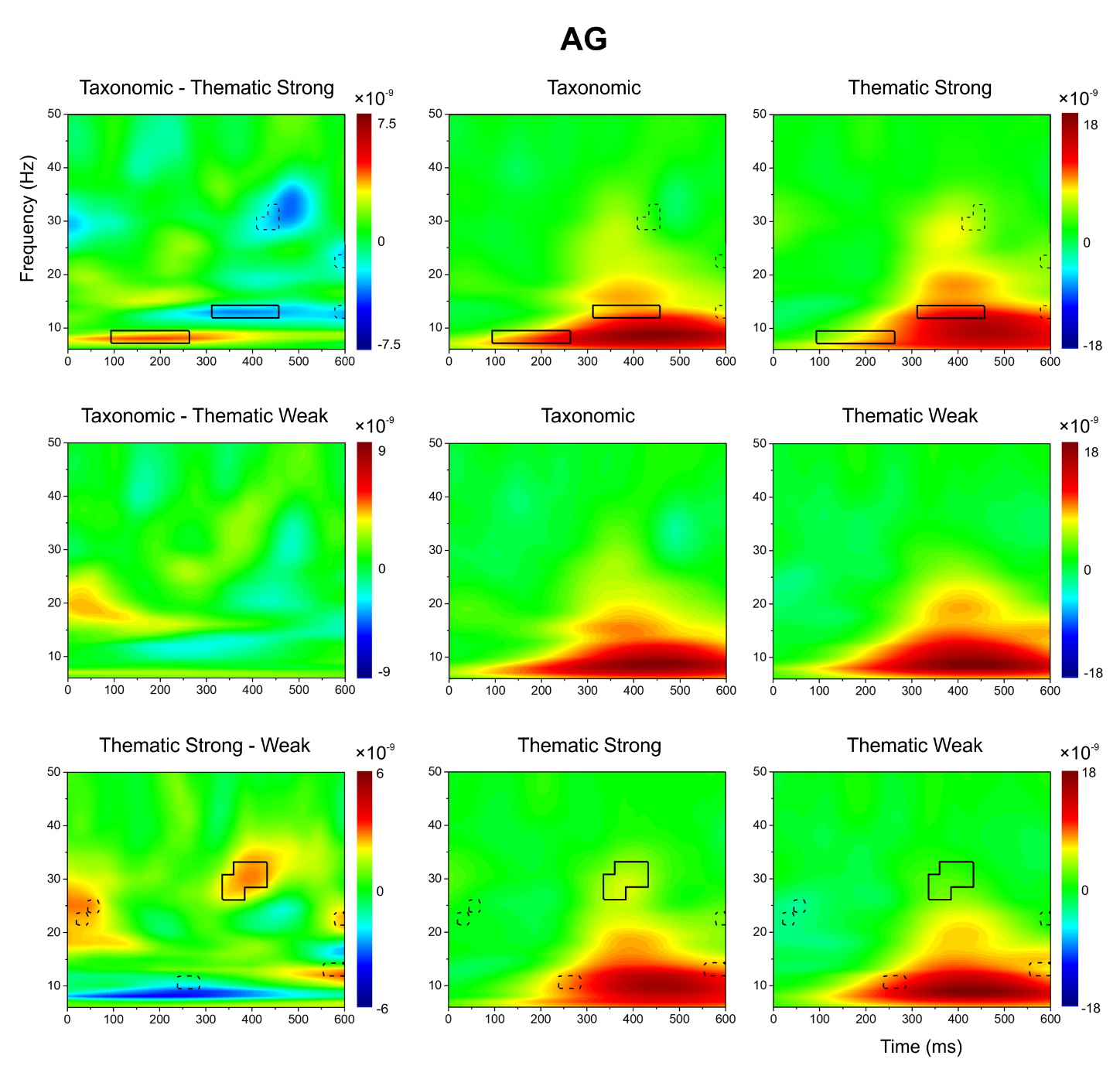


**Supplementary Figure S3:** Evoked power in AG. Difference plots (left-hand column)**:** Differences between taxonomic and strong thematic trials (TOP ROW), taxonomic and weak thematic trials (MIDDLE ROW) and strong vs. weak thematic trials (BOTTOM ROW). The black lines mark statistical contours fulfilling the following criteria: a) the difference between conditions reached *p* < 0.05; b) the region was also significantly different from baseline in at least one of the two contributing conditions at *p* < 0.01; c) the cluster had six or more contiguous pixels showing a significant difference. The plots for each condition (middle and right-hand column) show change for each trial type relative to “passive” baseline (before the onset of the trial).

Inferior frontal gyrus: Left IFG is thought to play an important role in semantic control (Badre, Poldrack, Paré-Blagoev, Insler, & Wagner, 2005; Noonan, Jefferies, Visser, & Lambon Ralph, 2013; Thompson-Schill, D’Esposito, Aguirre, & Farah, 1997). However, the Dual Hub and Controlled Semantic Cognition accounts do not make alternative predictions about the response at this site, and consequently it was not a primary target in our analysis.

Results: The results for IFG are shown in Figure S4. A strong evoked response to the presentation of the second word was observed within 100ms in the taxonomic condition and within 200ms in the thematic trials. By 300ms, this increase in evoked power extended from low frequencies up to 20Hz for all three conditions. However, there were few differences between conditions at this site. There were small differences between strong and weak thematic trials, but these were not systematic and did not reach the threshold of six contiguous tiles applied at other sites. There were also small taxonomic > weak thematic effects which did not reach the threshold of six contiguous tiles. When the taxonomic trials were contrasted with easier strong thematic trials, a stronger response was seen from 5-10Hz, from 100-350ms – this might reflect the greater controlled semantic retrieval demands of the harder taxonomic trials. However, within the first 50ms following the second word, there was also a stronger response to the easier strong thematic condition, which is not consistent with the role of this site in semantic control.

Discussion: While the results in ATL and pMTG could be readily linked to predictions of the controlled semantic cognition account, the findings for LIFG were unexpected. There were few consistent effects of strength of association. This is surprising since fMRI studies have consistently observed a stronger BOLD response in LIFG during the retrieval of weaker associations, plus responses to other manipulations of semantic control (Badre et al., 2005; Noonan et al., 2013; Thompson-Schill et al., 1997). These findings highlight the complex relationship between BOLD and MEG measures, which may reflect several factors. First, fMRI studies have demonstrated functional subdivisions within LIFG: anterior LIFG lies within the DMN (Davey et al., 2015; Yeo et al., 2011) and is specifically implicated in controlled retrieval from memory (Noonan et al., 2013). More posterior and dorsal portions of LIFG bordering inferior frontal sulcus fall within the frontoparietal network and are implicated in the process of selecting between competing alternatives within and beyond memory: for example, posterior LIFG contributes to phonological as well as semantic processing (Gough, Nobre, & Devlin, 2005). Our MEG analysis site was in mid-LIFG – perhaps because both of these processes were important for the semantic judgement task. In any case, MEG may lack the spatial resolution to separate these sources, and while a contribution to controlled semantic retrieval should result in stronger power changes for weak associations and taxonomic trials, the contribution of LIFG to language more generally might elicit the opposite effect.

**
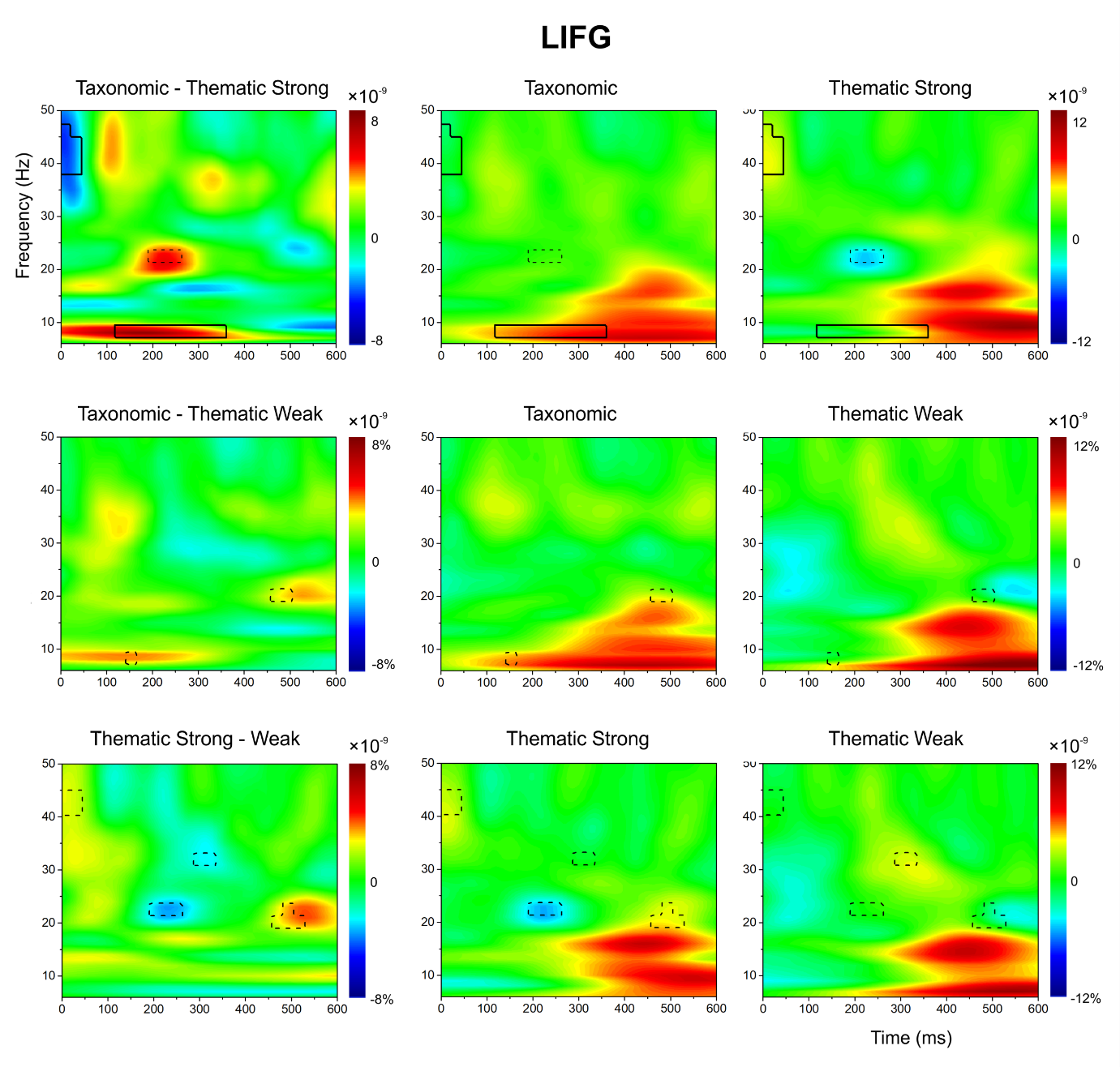
**

**Supplementary Figure S4:** Evoked power in IFG. Difference plots (left-hand column)**:** Differences between taxonomic and strong thematic trials (TOP ROW), taxonomic and weak thematic trials (MIDDLE ROW) and strong vs. weak thematic trials (BOTTOM ROW). The black lines mark statistical contours fulfilling the following criteria: a) the difference between conditions reached *p* < 0.05; b) the region was also significantly different from baseline in at least one of the two contributing conditions at *p* < 0.01; c) the cluster had six or more contiguous tiles showing a significant difference. The plots for each condition (middle and right-hand column) show signal change for each trial type relative to “passive” baseline.

List of presented items:

| abbey | monk | strong thematic |
| --- | --- | --- |
| apple | orchard | strong thematic |
| ashtray | cigarette | strong thematic |
| badge | officer | strong thematic |
| barn | hay | strong thematic |
| bayonet | battle | strong thematic |
| bedroom | dresser | strong thematic |
| bell | tower | strong thematic |
| bib | child | strong thematic |
| bible | hotel | strong thematic |
| biscuit | cheese | strong thematic |
| boat | harbour | strong thematic |
| boot | hike | strong thematic |
| car | garage | strong thematic |
| cart | pony | strong thematic |
| cat | vet | strong thematic |
| champagne | party | strong thematic |
| child | teddy | strong thematic |
| church | vicar | strong thematic |
| cloak | dagger | strong thematic |
| cow | milk | strong thematic |
| curtain | theatre | strong thematic |
| diver | tank | strong thematic |
| donkey | dung | strong thematic |
| farm | sheep | strong thematic |
| ferry | island | strong thematic |
| film | popcorn | strong thematic |
| finger | trigger | strong thematic |
| fireplace | ornament | strong thematic |
| flask | gin | strong thematic |
| forge | blacksmith | strong thematic |
| fort | infantry | strong thematic |
| garden | flower | strong thematic |
| goldfish | tank | strong thematic |
| grill | sausage | strong thematic |
| hair | brush | strong thematic |
| hammer | wall | strong thematic |
| helmet | miner | strong thematic |
| hen | cage | strong thematic |
| hook | worm | strong thematic |
| hoover | fluff | strong thematic |
| horse | harness | strong thematic |
| hotel | guest | strong thematic |
| hymn | organ | strong thematic |
| jail | cell | strong thematic |
| jug | water | strong thematic |
| lager | glass | strong thematic |
| lemonade | bottle | strong thematic |
| leopard | spot | strong thematic |
| lobster | fishtank | strong thematic |
| lorry | highway | strong thematic |
| menu | restaurant | strong thematic |
| monarch | throne | strong thematic |
| muzzle | dog | strong thematic |
| oven | cake | strong thematic |
| owl | barn | strong thematic |
| pike | river | strong thematic |
| pot | plant | strong thematic |
| pram | baby | strong thematic |
| pulpit | bible | strong thematic |
| pumpkin | halloween | strong thematic |
| python | jungle | strong thematic |
| rabbi | synagogue | strong thematic |
| rabbit | burrow | strong thematic |
| rat | maze | strong thematic |
| reed | flute | strong thematic |
| seaweed | sand | strong thematic |
| sheep | shear | strong thematic |
| shell | sand | strong thematic |
| shovel | garden | strong thematic |
| soil | garden | strong thematic |
| soldier | uniform | strong thematic |
| spider | web | strong thematic |
| squirrel | nut | strong thematic |
| stadium | ticket | strong thematic |
| suit | tailor | strong thematic |
| surgeon | scalpel | strong thematic |
| tea | mug | strong thematic |
| teeth | dentist | strong thematic |
| temple | pillar | strong thematic |
| theatre | actor | strong thematic |
| thread | stitch | strong thematic |
| toast | jam | strong thematic |
| tooth | comb | strong thematic |
| tractor | diesel | strong thematic |
| trail | forest | strong thematic |
| train | platform | strong thematic |
| tumbler | table | strong thematic |
| vase | tulip | strong thematic |
| veil | bride | strong thematic |
| waitress | restaurant | strong thematic |
| warrant | drugs | strong thematic |
| whale | plankton | strong thematic |
| whistle | train | strong thematic |
| worm | apple | strong thematic |
| abbey | headstone | weak thematic |
| apple | eve | weak thematic |
| ashtray | match | weak thematic |
| badge | competition | weak thematic |
| barn | rat | weak thematic |
| bayonet | trench | weak thematic |
| bedroom | duvet | weak thematic |
| bell | whistle | weak thematic |
| bib | porridge | weak thematic |
| bible | god | weak thematic |
| biscuit | tin | weak thematic |
| boat | rope | weak thematic |
| boot | trail | weak thematic |
| car | rust | weak thematic |
| cart | dust | weak thematic |
| cat | cushion | weak thematic |
| champagne | pool | weak thematic |
| child | seat | weak thematic |
| church | incense | weak thematic |
| cloak | friar | weak thematic |
| cow | meadow | weak thematic |
| curtain | kitchen | weak thematic |
| diver | coral | weak thematic |
| donkey | carrot | weak thematic |
| farm | tractor | weak thematic |
| ferry | engine | weak thematic |
| film | spool | weak thematic |
| finger | glove | weak thematic |
| fireplace | armchair | weak thematic |
| flask | hip | weak thematic |
| forge | anvil | weak thematic |
| fort | hilltop | weak thematic |
| garden | rake | weak thematic |
| goldfish | pond | weak thematic |
| grill | iron | weak thematic |
| hair | sink | weak thematic |
| hammer | auction | weak thematic |
| helmet | worker | weak thematic |
| hen | feather | weak thematic |
| hook | peg | weak thematic |
| hoover | cupboard | weak thematic |
| horse | saddle | weak thematic |
| hotel | soap | weak thematic |
| hymn | angel | weak thematic |
| jail | tattoo | weak thematic |
| jug | restaurant | weak thematic |
| lager | lime | weak thematic |
| lemonade | coke | weak thematic |
| leopard | hunt | weak thematic |
| lobster | butter | weak thematic |
| lorry | driver | weak thematic |
| menu | glass | weak thematic |
| monarch | law | weak thematic |
| muzzle | teeth | weak thematic |
| oven | blister | weak thematic |
| owl | snow | weak thematic |
| pike | military | weak thematic |
| pot | camp | weak thematic |
| pram | cot | weak thematic |
| pulpit | pastor | weak thematic |
| pumpkin | soup | weak thematic |
| python | tree | weak thematic |
| rabbi | prayer | weak thematic |
| rabbit | magician | weak thematic |
| rat | plague | weak thematic |
| reed | marsh | weak thematic |
| seaweed | rock | weak thematic |
| sheep | mountain | weak thematic |
| shell | turtle | weak thematic |
| shovel | shed | weak thematic |
| soil | tree | weak thematic |
| soldier | wound | weak thematic |
| spider | fly | weak thematic |
| squirrel | woodland | weak thematic |
| stadium | athletics | weak thematic |
| suit | lunch | weak thematic |
| surgeon | gloves | weak thematic |
| tea | china | weak thematic |
| teeth | sweets | weak thematic |
| temple | deity | weak thematic |
| theatre | paint | weak thematic |
| thread | crochet | weak thematic |
| toast | beans | weak thematic |
| tooth | sweets | weak thematic |
| tractor | mud | weak thematic |
| trail | desert | weak thematic |
| train | engine | weak thematic |
| tumbler | whiskey | weak thematic |
| vase | antique | weak thematic |
| veil | lace | weak thematic |
| waitress | napkin | weak thematic |
| warrant | detective | weak thematic |
| whale | communication | weak thematic |
| whistle | referee | weak thematic |
| worm | bait | weak thematic |
| abbey | lodge | taxonomic |
| apple | lime | taxonomic |
| ashtray | bin | taxonomic |
| badge | medal | taxonomic |
| barn | bunker | taxonomic |
| bayonet | axe | taxonomic |
| bedroom | dormitory | taxonomic |
| bell | rattle | taxonomic |
| bib | apron | taxonomic |
| bible | essay | taxonomic |
| biscuit | cracker | taxonomic |
| boat | coach | taxonomic |
| boot | slipper | taxonomic |
| car | wagon | taxonomic |
| cart | jeep | taxonomic |
| cat | mole | taxonomic |
| champagne | tea | taxonomic |
| child | baboon | taxonomic |
| church | mosque | taxonomic |
| cloak | coat | taxonomic |
| cow | bear | taxonomic |
| curtain | tablecloth | taxonomic |
| diver | runner | taxonomic |
| donkey | goat | taxonomic |
| farm | park | taxonomic |
| ferry | canoe | taxonomic |
| film | play | taxonomic |
| finger | claw | taxonomic |
| fireplace | hob | taxonomic |
| flask | tumbler | taxonomic |
| forge | oven | taxonomic |
| fort | bungalow | taxonomic |
| garden | pasture | taxonomic |
| goldfish | haddock | taxonomic |
| grill | furnace | taxonomic |
| hair | fur | taxonomic |
| hammer | stapler | taxonomic |
| helmet | crown | taxonomic |
| hen | robin | taxonomic |
| hook | scythe | taxonomic |
| hoover | mower | taxonomic |
| horse | elephant | taxonomic |
| hotel | monastery | taxonomic |
| hymn | tale | taxonomic |
| jail | tomb | taxonomic |
| jug | canister | taxonomic |
| lager | juice | taxonomic |
| lemonade | liquor | taxonomic |
| leopard | fox | taxonomic |
| lobster | scorpion | taxonomic |
| lorry | bicycle | taxonomic |
| menu | map | taxonomic |
| monarch | president | taxonomic |
| muzzle | mask | taxonomic |
| oven | aga | taxonomic |
| owl | chicken | taxonomic |
| pike | shark | taxonomic |
| pot | pail | taxonomic |
| pram | trolley | taxonomic |
| pulpit | dais | taxonomic |
| pumpkin | tomato | taxonomic |
| python | worm | taxonomic |
| rabbi | bishop | taxonomic |
| rabbit | beaver | taxonomic |
| rat | otter | taxonomic |
| reed | vine | taxonomic |
| seaweed | nettle | taxonomic |
| sheep | lama | taxonomic |
| shell | barnacle | taxonomic |
| shovel | spoon | taxonomic |
| soil | clay | taxonomic |
| soldier | policeman | taxonomic |
| spider | lice | taxonomic |
| squirrel | badger | taxonomic |
| stadium | auditorium | taxonomic |
| suit | robe | taxonomic |
| surgeon | butcher | taxonomic |
| tea | wine | taxonomic |
| teeth | beak | taxonomic |
| temple | college | taxonomic |
| theatre | saloon | taxonomic |
| thread | wire | taxonomic |
| toast | Pie | taxonomic |
| tooth | bone | taxonomic |
| tractor | tank | taxonomic |
| trail | street | taxonomic |
| train | submarine | taxonomic |
| tumbler | vase | taxonomic |
| vase | bucket | taxonomic |
| veil | cape | taxonomic |
| waitress | nurse | taxonomic |
| warrant | bill | taxonomic |
| whale | dinosaur | taxonomic |
| whistle | flute | taxonomic |
| worm | eel | taxonomic |
| aga | child | unrelated |
| apron | bell | unrelated |
| auditorium | trail | unrelated |
| axe | whistle | unrelated |
| baboon | champagne | unrelated |
| badger | abbey | unrelated |
| barnacle | tea | unrelated |
| beak | jail | unrelated |
| bear | tumbler | unrelated |
| beaver | pulpit | unrelated |
| bicycle | rat | unrelated |
| bill | bayonet | unrelated |
| bin | thread | unrelated |
| bishop | toast | unrelated |
| bone | spider | unrelated |
| bottle | soil | unrelated |
| bucket | cat | unrelated |
| bungalow | pram | unrelated |
| bunker | hymn | unrelated |
| butcher | web | unrelated |
| canister | train | unrelated |
| canoe | tooth | unrelated |
| cape | jug | unrelated |
| chicken | helmet | unrelated |
| claw | boot | unrelated |
| clay | monarch | unrelated |
| coach | donkey | unrelated |
| coat | reed | unrelated |
| college | pumpkin | unrelated |
| cracker | bible | unrelated |
| crown | biscuit | unrelated |
| dais | rabbit | unrelated |
| dinosaur | surgeon | unrelated |
| dormitory | apple | unrelated |
| eel | fireplace | unrelated |
| elephant | diver | unrelated |
| essay | sheep | unrelated |
| fishtank | hotel | unrelated |
| flute | pot | unrelated |
| fox | ashtray | unrelated |
| fur | badge | unrelated |
| furnace | squirrel | unrelated |
| glass | lorry | unrelated |
| goat | car | unrelated |
| haddock | church | unrelated |
| highway | whale | unrelated |
| hob | flask | unrelated |
| jeep | finger | unrelated |
| juice | horse | unrelated |
| lama | waitress | unrelated |
| lice | oven | unrelated |
| lime | tractor | unrelated |
| liquor | cow | unrelated |
| lodge | muzzle | unrelated |
| map | lager | unrelated |
| mask | barn | unrelated |
| medal | veil | unrelated |
| mole | temple | unrelated |
| monastery | nut | unrelated |
| mosque | hair | unrelated |
| mower | python | unrelated |
| nettle | uniform | unrelated |
| nurse | leopard | unrelated |
| otter | soldier | unrelated |
| oven | farm | unrelated |
| pail | teeth | unrelated |
| park | shell | unrelated |
| pasture | warrant | unrelated |
| pie | hammer | unrelated |
| play | boat | unrelated |
| policeman | goldfish | unrelated |
| president | vase | unrelated |
| rattle | lobster | unrelated |
| robe | owl | unrelated |
| robin | shovel | unrelated |
| runner | menu | unrelated |
| saloon | hen | unrelated |
| scorpion | hoover | unrelated |
| scythe | tailor | unrelated |
| shark | theatre | unrelated |
| slipper | grill | unrelated |
| spoon | ferry | unrelated |
| spot | stadium | unrelated |
| stapler | bedroom | unrelated |
| street | fort | unrelated |
| submarine | pike | unrelated |
| tablecloth | cart | unrelated |
| tale | forge | unrelated |
| tank | suit | unrelated |
| tea | worm | unrelated |
| tomato | curtain | unrelated |
| tomb | bib | unrelated |
| trolley | garden | unrelated |
| tumbler | rabbi | unrelated |
| vase | cloak | unrelated |
| vine | hook | unrelated |
| wagon | film | unrelated |
| wine | ticket | unrelated |
| wire | lemonade | unrelated |
| worm | seaweed | unrelated |

Badre, D., Poldrack, R. A., Paré-Blagoev, E. J., Insler, R. Z., & Wagner, A. D. (2005). Dissociable Controlled Retrieval and Generalized Selection Mechanisms in Ventrolateral Prefrontal Cortex. *Neuron, 47*(6), 907-918. doi:10.1016/j.neuron.2005.07.023

Bemis, D. K., & Pylkkänen, L. (2013). Basic Linguistic Composition Recruits the Left Anterior Temporal Lobe and Left Angular Gyrus During Both Listening and Reading. *Cerebral Cortex, 23*(8), 1859-1873. doi:10.1093/cercor/bhs170

Chan, A. M., Baker, J. M., Eskandar, E., Schomer, D., Ulbert, I., Marinkovic, K., . . . Halgren, E. (2011). First-Pass Selectivity for Semantic Categories in Human Anteroventral Temporal Lobe. doi:10.1523/jneurosci.3122-11.2011

Davey, J., Cornelissen, P. L., Thompson, H. E., Sonkusare, S., Hallam, G., Smallwood, J., & Jefferies, E. (2015). Automatic and Controlled Semantic Retrieval: TMS Reveals Distinct Contributions of Posterior Middle Temporal Gyrus and Angular Gyrus. doi:10.1523/JNEUROSCI.4705-14.2015

Gough, P. M., Nobre, A. C., & Devlin, J. T. (2005). Dissociating Linguistic Processes in the Left Inferior Frontal Cortex with Transcranial Magnetic Stimulation. *The Journal of Neuroscience, 25*(35), 8010.

Hanslmayr, S., Staudigl, T., & Fellner, M. C. (2012). Oscillatory power decreases and long-term memory: the information via desynchronization hypothesis. *Front Hum Neurosci, 6*, 74. doi:10.3389/fnhum.2012.00074

Humphreys, G. F., & Lambon Ralph, M. A. (2015). Fusion and Fission of Cognitive Functions in the Human Parietal Cortex. *Cereb Cortex, 25*(10), 3547-3560. doi:10.1093/cercor/bhu198

Murphy, C., Rueschemeyer, S. A., Watson, D., Karapanagiotidis, T., Smallwood, J., & Jefferies, E. (2017). Fractionating the anterior temporal lobe: MVPA reveals differential responses to input and conceptual modality. *Neuroimage, 147*, 19-31. doi:10.1016/j.neuroimage.2016.11.067

Noonan, K. A., Jefferies, E., Visser, M., & Lambon Ralph, M. A. (2013). Going beyond inferior prefrontal involvement in semantic control: evidence for the additional contribution of dorsal angular gyrus and posterior middle temporal cortex. *J Cogn Neurosci, 25*(11), 1824-1850. doi:10.1162/jocn_a_00442

Thompson-Schill, S. L., D’Esposito, M., Aguirre, G. K., & Farah, M. J. (1997). Role of left inferior prefrontal cortex in retrieval of semantic knowledge: A reevaluation. In *Proc Natl Acad Sci U S A* (Vol. 94, pp. 14792-14797).

Yeo, B. T., Krienen, F. M., Sepulcre, J., Sabuncu, M. R., Lashkari, D., Hollinshead, M., . . . Buckner, R. L. (2011). The organization of the human cerebral cortex estimated by intrinsic functional connectivity. *J Neurophysiol, 106*(3), 1125-1165. doi:10.1152/jn.00338.2011
